# *SNHG16*/*miR‐605‐3p*/*TRAF6*/NF‐κB feedback loop regulates hepatocellular carcinoma metastasis

**DOI:** 10.1111/jcmm.15399

**Published:** 2020-05-20

**Authors:** Yi‐Lin Hu, Ying Feng, Yu‐Yan Chen, Jia‐Zhou Liu, Yang Su, Peng Li, Hua Huang, Qin‐Sheng Mao, Wan‐Jiang Xue

**Affiliations:** ^1^ Department of General Surgery Affiliated Hospital of Nantong University Nantong China; ^2^ Research Center of Clinical Medicine Affiliated Hospital of Nantong University Nantong China; ^3^ Department of Surgery The Affiliated Huaian No. 1 People's Hospital of Nanjing Medical University Huaian China; ^4^ Department of Pathology Affiliated Hospital of Nantong University Nantong China

**Keywords:** feedback loop, hepatocellular carcinoma, metastasis, *miR‐605‐3p*, NF‐κB signalling

## Abstract

The mechanism by which *miR‐605‐3p* regulates hepatocellular carcinoma (HCC) metastasis has not been clarified. In this study, we found that *miR‐605‐3p* was down‐regulated in HCC and that low *miR‐605‐3p* expression was associated with tumour thrombus and tumour satellites. HCC patients with low *miR‐605‐3p* expression showed shorter overall survival and disease‐free survival after surgery. Overexpression of *miR‐605‐3p* inhibited epithelial‐mesenchymal transition and metastasis of HCC through NF‐κB signalling by directly inhibiting expression of *TRAF6*, while silencing of *miR‐605‐3p* had the opposite effect. We also found that *SNHG16* directly bound to *miR‐605‐3p* as a competing endogenous RNA. Mechanistically, high expression of *SNHG16* promoted binding to *miR‐605‐3p* and inhibited its activity, which led to up‐regulation of *TRAF6* and sustained activation of the NF‐κB pathway, which in turn promoted epithelial‐mesenchymal transition and metastasis of HCC. TRAF6 increased *SNHG16* promoter activity by activating NF‐κB, thereby promoting the transcriptional expression of *SNHG16* and forming a positive feedback loop that aggravated HCC malignancy. Our findings reveal a mechanism for the sustained activation of the *SNHG16*/*miR‐605‐3p*/*TRAF6*/NF‐κB feedback loop in HCC and provide a potential target for a new HCC treatment strategy.

## 1 INTRODUCTION

Hepatocellular carcinoma (HCC) is the third leading cause of cancer‐related deaths worldwide, with nearly half of all cases occurring in China.^1^ The recurrence and metastasis rates of HCC after surgery remain high, and the prognosis of HCC patients is poor.^2^ Epithelial‐mesenchymal transition (EMT) is an important cause of HCC metastasis.^3^ However, its underlying mechanism remains unclear. Therefore, exploring the molecular mechanism of EMT in HCC is not only critical for the further understanding of metastasis in HCC, but may also provide clues towards identifying early diagnostic markers of HCC and new therapeutic targets.

Non‐coding RNAs are a class of RNAs that lack protein‐coding ability and are involved in regulating several cellular processes, including malignant biological activity in cancers.^4^ These molecules are classified as short non‐coding RNAs or long non‐coding RNAs (lncRNAs) according to their length. MicroRNAs (miRNAs) are a class of short non‐coding RNAs of 21‐23 bp in length that mediate RNA‐induced silencing complex (RISC) formation at the 3′‐untranslated region (UTR) of target genes, which results in target gene degradation or translational inhibition. Previous studies have shown that miRNAs are involved in the regulation of cellular processes such as organ formation, fat metabolism, cell proliferation and apoptosis.^5^ Furthermore, studies have shown that abnormal miRNA regulation may be a critical factor leading to HCC metastasis.^6^

LncRNAs are non‐coding RNAs of more than 200 bp in length that regulate chromatin modification, transcription, miRNAs, mRNA stability, protein function and other important regulatory functions.^7^, ^8^ LncRNAs exert important functions as critical regulators of various cell processes, and their abnormal expression may play an important role in cancer metastasis.^9^

Competitive endogenous RNAs (ceRNAs) represent a novel gene expression regulatory mechanism for lncRNA‐miRNA‐mRNA interactions.^10^ Recent studies showed that ceRNAs are involved in regulating various cellular activities such as HCC metastasis.^11^ LncRNAs act as miRNA sponges and competitively bind to common miRNAs through the miRNA response element, which attenuates the miRNA‐mediated inhibition of target genes and increases target gene expression. The target gene in turn regulates the progression of cancer metastasis. miRNAs are the core RNA molecules in the ceRNA hypothesis.^10^

*miR‐605‐3p* is a recently discovered miRNA with tumour suppressor functions.^12^, ^13^, ^14^ Overexpression of *miR‐605‐3p* inhibits the migration and invasion of bladder cancer and glioma cells.^13^, ^14^ However, its expression and function in HCC have not been reported.

In this study, we revealed a tumour‐suppressive function of *miR‐605‐3p* in human HCC for the first time. Mechanistically, *miR‐605‐3p* targets *TRAF6*, a signal transducer in the NF‐κB signalling pathway that has a crucial role in activating NF‐κB signalling to suppress *TRAF6* expression and thereby repress NF‐κB signalling. We further found that *SNHG16* is overexpressed in HCC cells and can directly bind to and affect *miR‐605‐3p* function, which leads to up‐regulation of *TRAF6* and continuous NF‐κB activation in HCC. In turn, TRAF6 up‐regulates *SNHG16* expression via NF‐κB/p65.

## 2 MATERIALS AND METHODS

### 2.1 Cell lines, cell culture and reagents

HCCLM3, MHCC97L and MHCC‐97H cell lines were gifts from the Liver Cancer Institute, ZhongShan Hospital. A normal hepatocyte cell line (L02) and two HCC cell lines (Hep3B and HepG2) were purchased from GeneChem. All cells were cultured in Dulbecco's modified Eagle's medium containing 10% foetal bovine serum with 100 U/mL penicillin and 100 mg/mL streptomycin in a humidified incubator at 37°C containing 5% CO_2_. SN‐50 was purchased from MedChem Express.

### 2.2 Patients and tissue samples

All HCC tissues and matched adjacent normal tissues were collected from the Affiliated Hospital of Nantong University. A total of 78 HCC tumour samples and matched adjacent normal tissues were obtained between 2004 and 2009 at the Department of General Surgery, and a panel of 16 fresh HCC cancer tissues and adjacent normal tissues, including eight metastasis‐free tissues and eight intrahepatic metastasis tissues, were obtained between 2012 and 2018. All patients with clear HCC pathology had never received neoadjuvant chemotherapy, radiation therapy or immunotherapy before surgery. The demographic and clinical characteristics of the HCC patients are shown in Table 1. Follow‐up was completed by August 2015. Approval was obtained from the Human Research Ethics Committee of Nantong University Affiliated Hospital, and written informed consent was obtained from each patient.

**TABLE 1:** Relationships between *miR‐605‐3p* expression and clinicopathological characteristics of HCC patients

Clinicopathological characteristics	n	Low expression	High expression	*χ^2^*	*P* value
Total	78	39	39		
Gender				1.156	.282
Male	60	28	32		
Female	18	11	7		
Age (years)				2.519	.112
≤54	41	17	24		
>54	37	22	15		
Grade of differentiation				1.472	.225
Low	25	15	10		
High‐Middle	53	24	29		
Tumour diameter (cm)				2.540	.111
≤5	43	25	18		
>5	35	14	21		
Liver function (Child‐Pugh stage)				2.063	.151
A	63	29	34		
B or C	15	10	5		
Hepatocirrhosis				2.077	.150
Absent	26	16	10		
Present	52	23	29		
HBV infection				3.284	.070
Absent	38	15	23		
Present	40	24	16		
Tumour thrombus				6.303	.012*
Absent	66	29	37		
Present	12	10	2		
AFP (ng/ml)				0.825	.364
≤20	42	23	19		
>20	36	16	20		
BCLC stage				3.193	.074
A	57	32	25		
B, C, or D	21	7	14		
Envelope				3.391	.066
Absent	46	12	20		
Present	32	27	19		
Tumour satellite				5.032	.025*
Absent	62	28	34		
Present	16	12	4		

Abbreviations: AFP, serum alpha fetoprotein; BCLC, Barcelona Clinic Liver Cancer; HBV, hepatitis B virus;

* *P* < .05.

### 2.3 Online bioinformatics analysis

Putative *miR‐605‐3p* target genes were predicted by TargetScan (http://www.targetscan.org/vert_72/). Putative lncRNAs targeting *miR‐605‐3p* were predicted by Starbase (http://starbase.sysu.edu.cn/). Putative transcription factors that regulate *SNHG16* expression were predicted by PROMO (http://alggen.lsi.upc.es/cgi‐bin/promo_v3/promo/promoinit.cgi?dirDB=TF_8.3), JASPAR (jaspar.genereg.net/) and LASAGNA (https://biogrid‐lasagna.engr.uconn.edu/lasagna_search/). The GEPIA database (http://gepia2.cancer‐pku.cn/) was used to analyse the expression correlation between *SNHG16* and *RELA* (NF‐κB/p65), *SNHG16* expression and the prognostic significance of *SNHG16* expression in the liver hepatocellular carcinoma data set of TCGA database.

### 2.4 Chromatin immunoprecipitation (ChIP) assays

Cells were cross‐linked with 1% formaldehyde and quenched in glycine solution. ChIP assays were performed using a Pierce Magnetic ChIP Kit (Thermo Fisher Scientific, Waltham, MA, USA), according to the manufacturer's protocol. Anti‐p65 antibody and normal IgG (MultiSciences) were used for immunoprecipitation. ChIP‐enriched DNA samples were analysed by qRT‐PCR to quantify the putative p65‐binding sites in the *SNHG16* promoter region. The data are shown as relative enrichment normalized to control IgG. Primer sequences for ChIP assays were as follows: forward, 5′‐CCTGGTAAGTGCTATGAAGT‐3′; reverse, 5′‐TCTATCCCTGCAAACATAGT‐3′.

### 2.5 Luciferase reporter assays

For NF‐κB luciferase assays, 3 × 10^4^ HCC cells/well were seeded in 48‐well plates and cultured for 24 hours. A pNF‐κB‐luciferase plasmid (GeneChem), control luciferase plasmid, pRL‐TK *Renilla* plasmid (GeneChem) and *miR‐605‐3p* agomir or *miR‐605‐3p* agomir control were cotransfected into HCC cells using Lipofectamine 3000 (Invitrogen). After 48 hours, firefly and *Renilla* luciferase activities were measured with a Dual Luciferase Reporter Assay Kit (Beyotime).

To observe the interactions between *miR‐605‐3p* and *TRAF6*, the 3′‐UTR of *TRAF6* and the 3′‐UTR containing a mutated putative binding site were cloned into the pGL3 luciferase reporter plasmid (GeneChem). TRAF6‐WT or TRAF6‐MUT was cotransfected with *miR‐605‐3p* agomir or *miR‐605‐3p* agomir control. To observe the interactions between *SNHG16* and *miR‐605‐3p*, *SNHG16* and the mutated putative binding site of *miR‐605‐3p* in *SNHG16* were cloned into the pGL3 luciferase reporter plasmid (GeneChem). *SNHG16*‐WT or *SNHG16*‐MUT was cotransfected with *miR‐605‐3p* agomir or *miR‐605‐3p* agomir control. At 48 hours post‐transfection, a Dual Luciferase Assay (Beyotime) was used to determine the luciferase reporter activities according to the manufacturer's instructions.

Putative binding sites of transcription factors were analysed using the JASPAR, PROMO and LASAGNA databases (assembly Grch38/hg38). The predictive *SNHG16* promoter region was chr17:76555764‐76557863. The promoter region was chr17:76555900‐76555909, which included the binding site for NF‐κB/p65 (GGGAAATTCC). The wild‐type *SNHG16* promoter and a promoter with mutated NF‐κB‐binding sites were constructed by GeneChem. *SNHG16*‐WT or *SNHG16*‐MUT was cotransfected with pcDNA3.1 vector or pcDNA3.1 NF‐κB/p65 (GeneChem). A dual‐luciferase reporter assay was conducted to measure the functional NF‐κB/p65 binding site in the *SNHG16* promoter. Firefly luciferase activity was normalized to *Renilla* luciferase activity and presented as relative luciferase activity.

The details for RNA extraction, qRT‐PCR, RNA fluorescence in situ hybridization, subcellular fractionation, Western blotting, immunohistochemistry, cell transfection, lentivirus production and transduction, wound healing assay, Matrigel invasion assay, immunofluorescence, RNA immunoprecipitation assays, in vivo animal experiments and statistical analyses are described in the Supplementary Materials.

## 3 RESULTS

### 3.1 *miR‐605‐3p* is down‐regulated in HCC and is associated with poor prognosis

**FIGURE 1:**
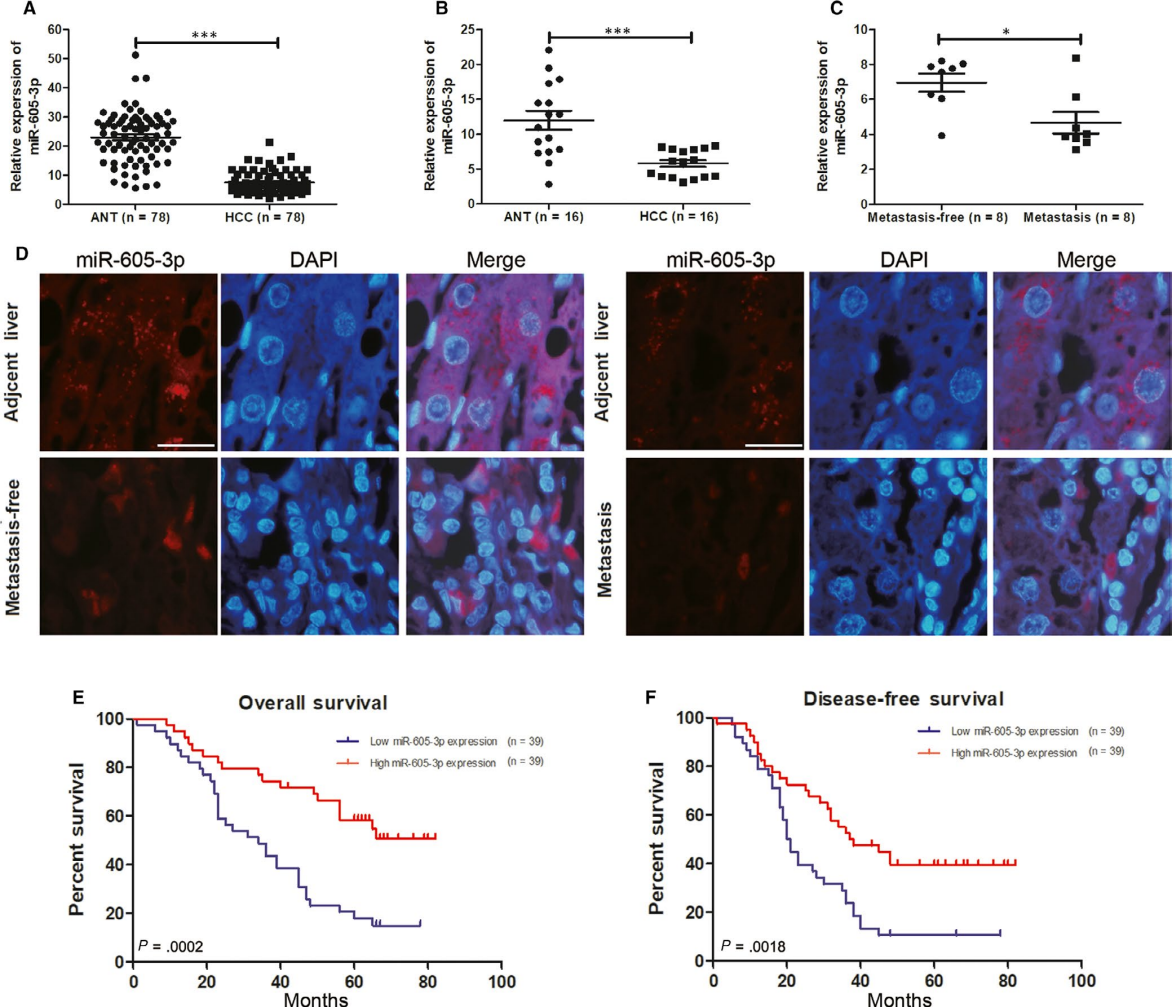
Low expression of *miR‐605‐3p* is related to poor prognosis of hepatocellular carcinoma (HCC) patients. A, *miR‐605‐3p* expression was detected using real‐time qPCR in 78 HCC tissues and matched adjacent normal tissues. *miR‐605‐3p* expression was normalized to *U6*. B, Relative expression of *miR‐605‐3p* was detected using real‐time qPCR in 16 HCC tissues and matched adjacent normal tissues. C, Relative expression of *miR‐605‐3p* was detected using real‐time qPCR in 16 HCC tissues with or without intrahepatic metastasis. D, Typical image of *miR‐605‐3p* expression detected by fluorescence in situ hybridization in 16 HCC tissues with or without intrahepatic metastasis and their adjacent normal tissues (scale bar, 25 μm). E and F, Correlation between *miR‐605‐3p* expression and overall survival (E) and disease‐free survival (F) by Kaplan‐Meier analysis of HCC patients with high or low *miR‐605‐3p* expression. *P < .05, ***P < .001

To investigate the role of *miR‐605‐3p* in HCC, we first detected the expression levels of *miR‐605‐3p* in 78 HCC and paired adjacent normal tissues. The results showed that *miR‐605‐3p* expression was down‐regulated in HCC tissues (Figure 1A). Analysis of the clinicopathological characteristics showed that low *miR‐605‐3p* expression was positively associated with tumour thrombus and tumour satellites in the 78 HCC patients (Table 1). qRT‐PCR (Figure 1B, C) and fluorescence in situ hybridization (Figure 1D) data indicated that the relative expression of *miR‐605‐3p* was significantly lower in the intrahepatic metastasis group compared with the intrahepatic metastasis‐free metastasis group. Kaplan‐Meier analysis showed that HCC patients with low *miR‐605‐3p* expression had worse overall survival (OS) and disease‐free survival (DFS) rates than those with high *miR‐605‐3p* expression (Figure 1E, F). Multivariate analysis also showed that low expression of *miR‐605‐3p* was an independent predictor of OS and DFS (Table 2). Taken together, these results suggest that *miR‐605‐3p* may protect against HCC.

**TABLE 2:** Univariate and multivariable analyses of OS and DFS in HCC patients

Variable	OS			DFS		
	Univariate analysis	Multivariable analysis		Univariate analysis	Multivariable analysis	
	*P*>|z|	*P*>|z|	HR(95%CI)	*P*>|z|	*P*>|z|	HR(95%CI)
miR‐605‐3p expression						
Low (n = 39) vs. high (n = 39)	<.001*	.002*	0.383 (0.210‐0.702)	.002*	.005*	0.452 (0.260‐0.783)
Gender						
Male (n = 60) vs. female (n = 18)	.131			.205		
Age (years)						
≤54 (n = 41) vs. >54 (n = 37)	.833			.537		
Grade of differentiation						
Low (n = 39) vs. middle‐high (n = 39)	.101			.148		
Tumour diameter (cm)						
≤5 (n = 43) vs. >5 (n = 35)	.200			.194		
Liver function (Child‐Pugh stage)						
A (n = 63) vs. B or C (n = 15)	.182			.507		
Hepatocirrhosis						
Absent (n = 26) vs. present (n = 52)	.425			.176		
Hepatitis B virus						
Absent (n = 38) vs. present (n = 40)	0.177			.553		
Tumour thrombus						
Absent (n = 66) vs. present (n = 12)	.011*			.008*		
AFP (ng/ml)						
≤20 (n = 42) vs. >20 (n = 36)	.060			.107		
BCLC stage						
I (n = 57) vs. II, III, or IV (n = 21)	.151			.763		
Envelope						
Absent (n = 46) vs. present (n = 32)	.189			.168		
Tumour satellite						
Absent (n = 62) vs. present (n = 16)	.068			.069		

* *P* < .05.

### 3.2 *miR‐605‐3p* suppresses HCC cell metastasis in vitro and in vivo

**FIGURE 2:**
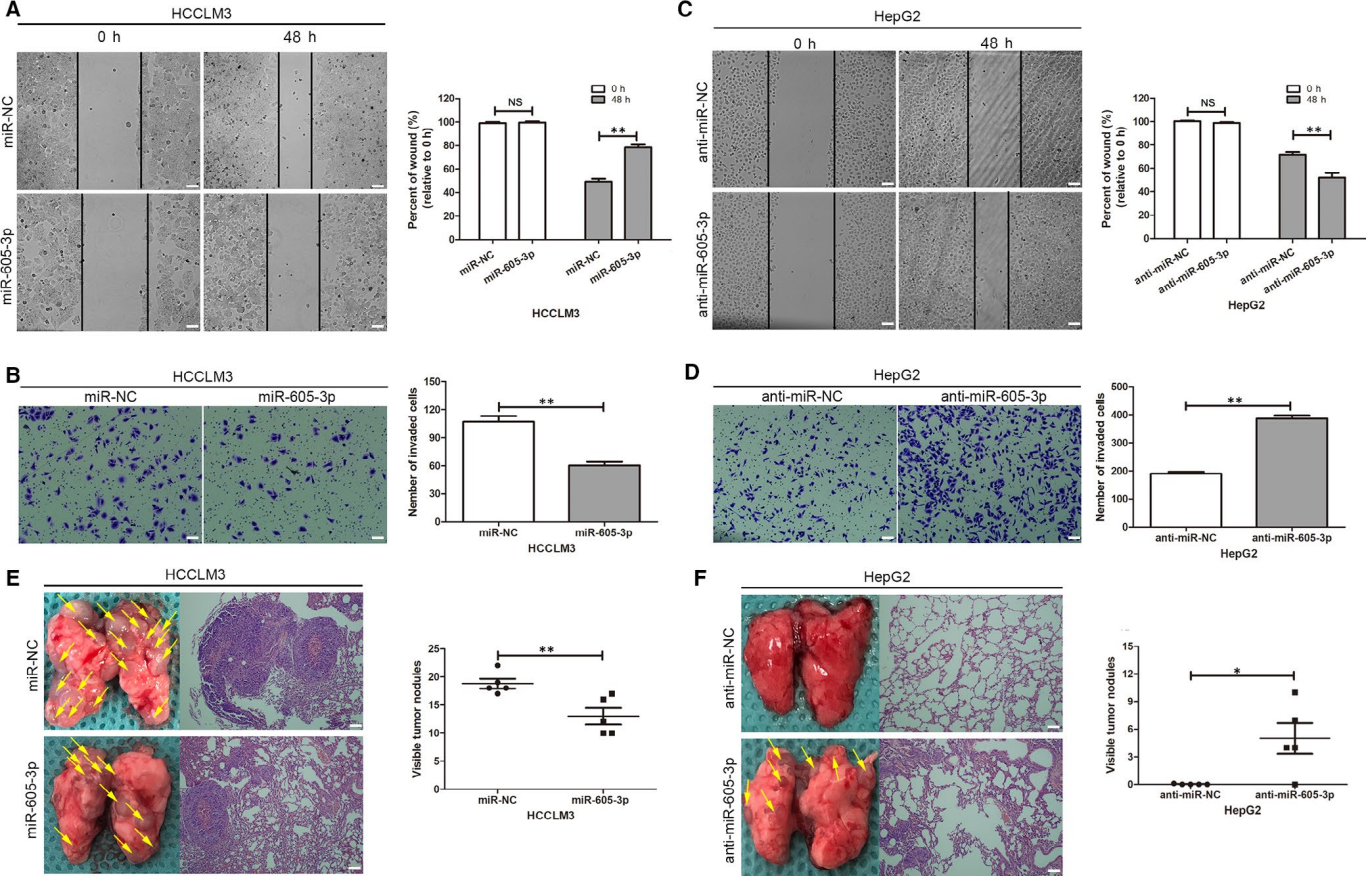
*miR‐605‐3p* inhibits HCC cell migration and invasion in vitro and in vivo. A–D, The effects of *miR‐605‐3p* overexpression in HCCLM3 cells (A, B) and *miR‐605‐3p* silencing in HepG2 cells (C, D) on migration and invasion were analysed by wound healing and Matrigel transwell assays. (scale bar, 100 μm) E and F, *miR‐605‐3p* inhibited HCC cell metastasis in vivo. (Left) Representative bright‐field images of lungs. (Right) Haematoxylin and eosin (H&E) staining of lung serial sections. Arrows indicate lung lesions. (Panel) Numbers of nodules on the lungs of mice (n = 5 per group) at 6 weeks after tail vein injection of HCCLM3/miR‐NC or HCCLM3/*miR‐605‐3p* cells (scale bar, 25 μm) (E) and HepG2/anti‐miR‐NC or HepG2/anti‐*miR‐605‐3p* cells (F). *P < .05, **P < .01. NS: no significance

qRT‐PCR analyses showed that *miR‐605‐3p* expression was highest in HepG2 cells and lowest in HCCLM3 cells among the five HCC cell lines examined in this study (Figure S1A). Therefore, HCCLM3 and HepG2 cells were chosen for subsequent experiments. We confirmed the efficiency of overexpression or silencing of *miR‐605‐3p* in both HCC cell lines (Figure S1B). Wound healing and Matrigel invasion assays showed that overexpression of *miR‐605‐3p* specifically suppressed HCCLM3 cell migration and invasion (Figure 2A, B), while *miR‐605‐3p* silencing led to increased HepG2 cell migration and invasion (Figure 2C, D).

We further investigated whether *miR‐605‐3p* expression regulated the metastatic ability of HCC cells in vivo. We found that the number and size of metastatic colonies on the lung surface of mice were largely decreased in the HCCLM3/*miR‐605‐3p* group (Figure 2E). We also detected metastatic colonies in the HepG2/anti‐*miR‐605‐3p* group, while no metastatic colonies were found in the HepG2/anti‐miR‐NC group (Figure 2F). These results indicated that *miR‐605‐3p* inhibited the metastatic ability of HCC cells in vitro and in vivo.

### 3.3 *miR‐605‐3p* inhibits EMT in HCC cells

**FIGURE 3:**
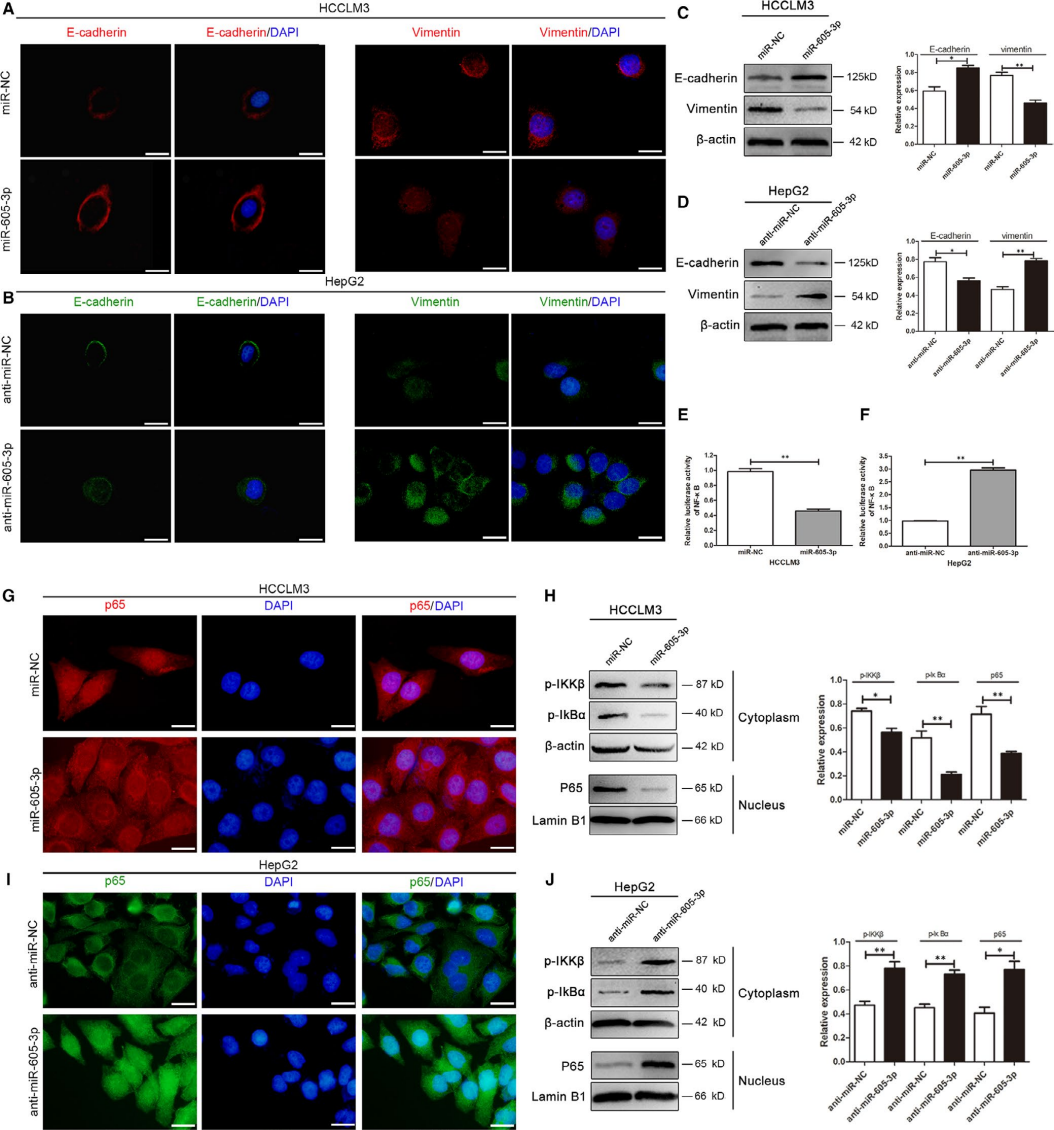
*miR‐605‐3p* inhibits epithelial‐mesenchymal transition (EMT) in HCC cells and attenuates NF‐κB signalling activation. A–D, Immunofluorescence and Western blotting were conducted to examine the effects of *miR‐605‐3p* overexpression on EMT in HCCLM3 cells (scale bar, 25 μm) (A, C) and *miR‐605‐3p* silencing on EMT in HepG2 cells (scale bar, 25 μm) (B, D). E–J, NF‐κB luciferase reporter activity, immunofluorescence and Western blotting analyses were performed to detect the effects of *miR‐605‐3p* overexpression on NF‐κB signalling activation in HCCLM3 cells (scale bar, 25 μm) (E, G, H) and *miR‐605‐3p* silencing on NF‐κB signalling activation in HepG2 cells (scale bar, 25 μm) (F, I, J). *P < .05, **P < .01

EMT is a critical process involved in cancer metastasis.^15^ To investigate whether *miR‐605‐3p* regulates EMT in HCC cells, immunofluorescence and Western blotting assays were conducted. The results showed that up‐regulation of *miR‐605‐3p* in HCCLM3 cells increased E‐cadherin expression and decreased vimentin expression (Figure 3A, C). Conversely, down‐regulation of *miR‐605‐3p* in HepG2 cells decreased E‐cadherin expression and increased vimentin expression (Figure 3B, D).

### 3.4 *miR‐605‐3p* inhibits NF‐κB activation in HCC

NF‐κB is an important pathway that regulates EMT and metastasis in HCC.^16^ Thus, we examined whether *miR‐605‐3p* regulates activation of the NF‐κB pathway. Luciferase reporter assays indicated that NF‐κB‐induced activity was reduced in *miR‐605‐3p*‐overexpressing cells (Figure 3E) but increased in *miR‐605‐3p*‐silenced cells (Figure 3F). Immunofluorescence assays showed that NF‐κB/p65 nuclear expression was decreased in *miR‐605‐3p*‐overexpressing cells (Figure 3G) but increased in *miR‐605‐3p*‐silenced cells (Figure 3I). Western blotting analyses showed that up‐regulation of *miR‐605‐3p* expression reduced the phosphorylation of IKK‐β and IκBα (Figure 3H) but increased their phosphorylation in *miR‐605‐3p*‐silenced cells (Figure 3J). Additionally, *miR‐605‐3p* up‐regulation decreased nuclear p65 expression (Figure 3H), while *miR‐605‐3p* down‐regulation increased nuclear p65 expression (Figure 3J).

### 3.5 *miR‐605‐3p* targets TRAF6 in HCC

**FIGURE 4:**
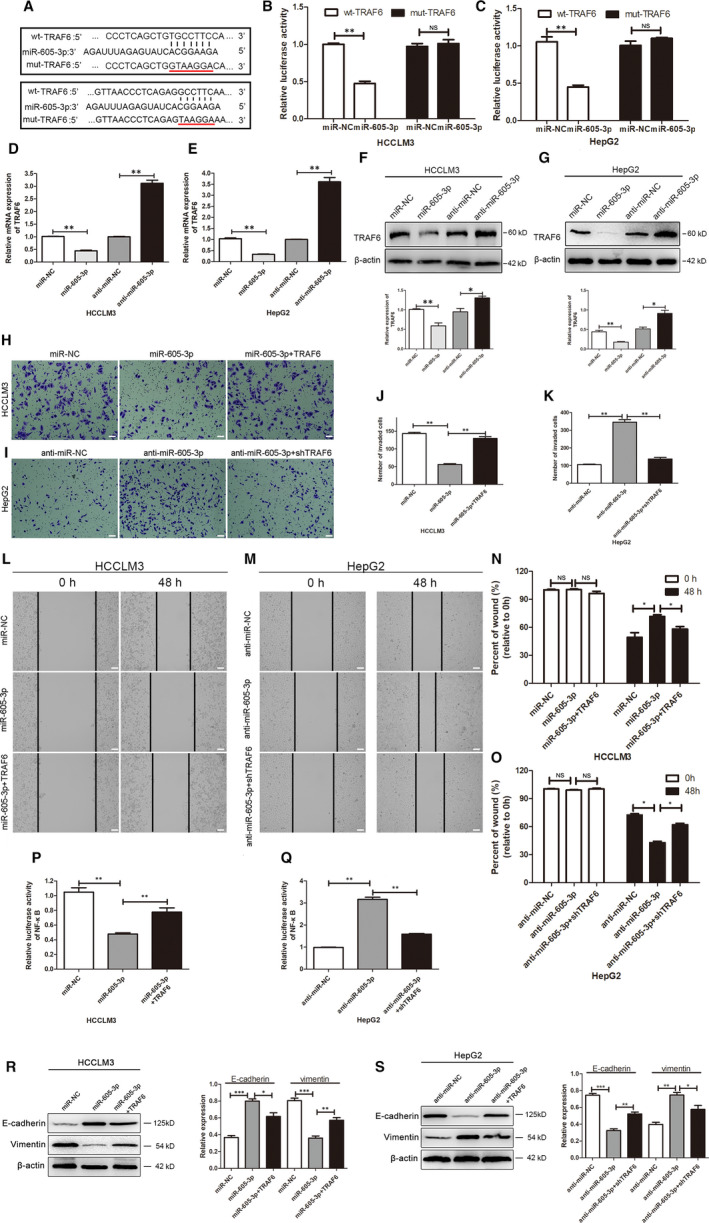
*TRAF6* is a direct target of *miR‐605‐3p* and affects the function of *miR‐605‐3p*. A, The potential *miR‐605‐3p*‐binding site in the *TRAF6* 3′‐untranslated region was predicted by TargetScan, as well as mutated nucleotides in the seed sequence of *miR‐605‐3p*. B, C, Dual‐luciferase reporter assays were performed to detect the effects of *miR‐605‐3p* overexpression in HCCLM3 cells (B) and *miR‐605‐3p* silencing in HepG2 cells (C) on the luciferase activity of reporters driven by the WT and MUT *TRAF6* 3′‐untranslated region. D–G, mRNA and protein levels of TRAF6 in *miR‐605‐3p*‐overexpressing HCCLM3 cells (D, F) and *miR‐605‐3p*‐silenced HepG2 cells (E, G). H–O, Matrigel transwell and wound healing assays were performed to analyse the invasion capacity of *miR‐605‐3p*‐overexpressing HCCLM3 cells after TRAF6 transfection (H, J, L, N) and *miR‐605‐3p*‐silenced HepG2 cells after shTRAF6 transfection (I, K, M, O) (scale bar, 100 μm). P, Q, Luciferase reporter activity assays were performed to analyse the effects of TRAF6 transfection in *miR‐605‐3p*‐overexpressing HCCLM3 cells (P) and the effects of shTRAF6 transfection on NF‐κB signalling in *miR‐605‐3p*‐silenced HepG2 cells. (Q). R, S, Western blotting analyses were performed to analyse the EMT biomarkers E‐cadherin and vimentin in *miR‐605‐3p*‐overexpressing HCCLM3 cells after TRAF6 transfection (R) and in *miR‐605‐3p*‐silenced HepG2 cells after shTRAF6 transfection (S). *P < .05, **P < .01. NS: no significance

We next predicted the target genes of *miR‐605‐3p* by TargetScan. Among the identified genes, *TRAF6* was identified as a gene of interest, as previous studies showed that *TRAF6* regulates the activation of NF‐κB signalling and affects NF‐κB‐mediated EMT in carcinogenesis and cancer development.^17^ Therefore, we first investigated whether *miR‐605‐3p* regulates *TRAF6* through binding to the 3′‐UTR of *TRAF6*. WT‐TRAF6 or MUT‐TRAF6 3′‐UTR luciferase reporter vectors (Figure 4A) along with *miR‐605‐3p* or miR‐NC were cotransfected into HCCLM3 and HepG2 cells. Activity of the WT‐TRAF6 3′‐UTR vector was significantly down‐regulated in the *miR‐605‐3p* group compared with the miR‐NC group in HCCLM3 and HepG2 cells (Figure 4B, C). Conversely, *miR‐605‐3p* had no impact on the MUT‐TRAF6 3′‐UTR reporter. Furthermore, qPCR and Western blot assays showed that *miR‐605‐3p* overexpression down‐regulated *TRAF6* expression, while *miR‐605‐3p* knockdown up‐regulated *TRAF6* expression in HCCLM3 (Figure 4D, F) and HepG2 cells (Figure 4E,G).

We next investigated whether *TRAF6* is involved in the effects of *miR‐605‐3p* on HCC cell migration. The suppressive effects of *miR‐605‐3p* overexpression on cell migration and invasion were partly abolished by TRAF6 overexpression in HCCLM3 cells (Figure 4H, J, L, N). Furthermore, silencing of *TRAF6* blocked the effects of down‐regulated TRAF6 expression on the migration and invasion of *miR‐605‐3p*‐silenced HepG2 cells (Figure 4I, K, M, O). We also found that exogenous expression of TRAF6 abolished *miR‐605‐3p*‐induced inhibition of NF‐κB‐mediated EMT in HCCLM3 cells (Figure 4P, R), while silencing of *TRAF6* blocked *miR‐605‐3p*‐induced activation of NF‐κB‐mediated EMT in HepG2 cells (Figure 4Q, S). The efficiency of *TRAF6* overexpression or silencing in HCC cell lines was confirmed (Figure S1C, D).

### 3.6 Reciprocal negative regulation between *miR‐605‐3p* and *SNHG16*

**FIGURE 5:**
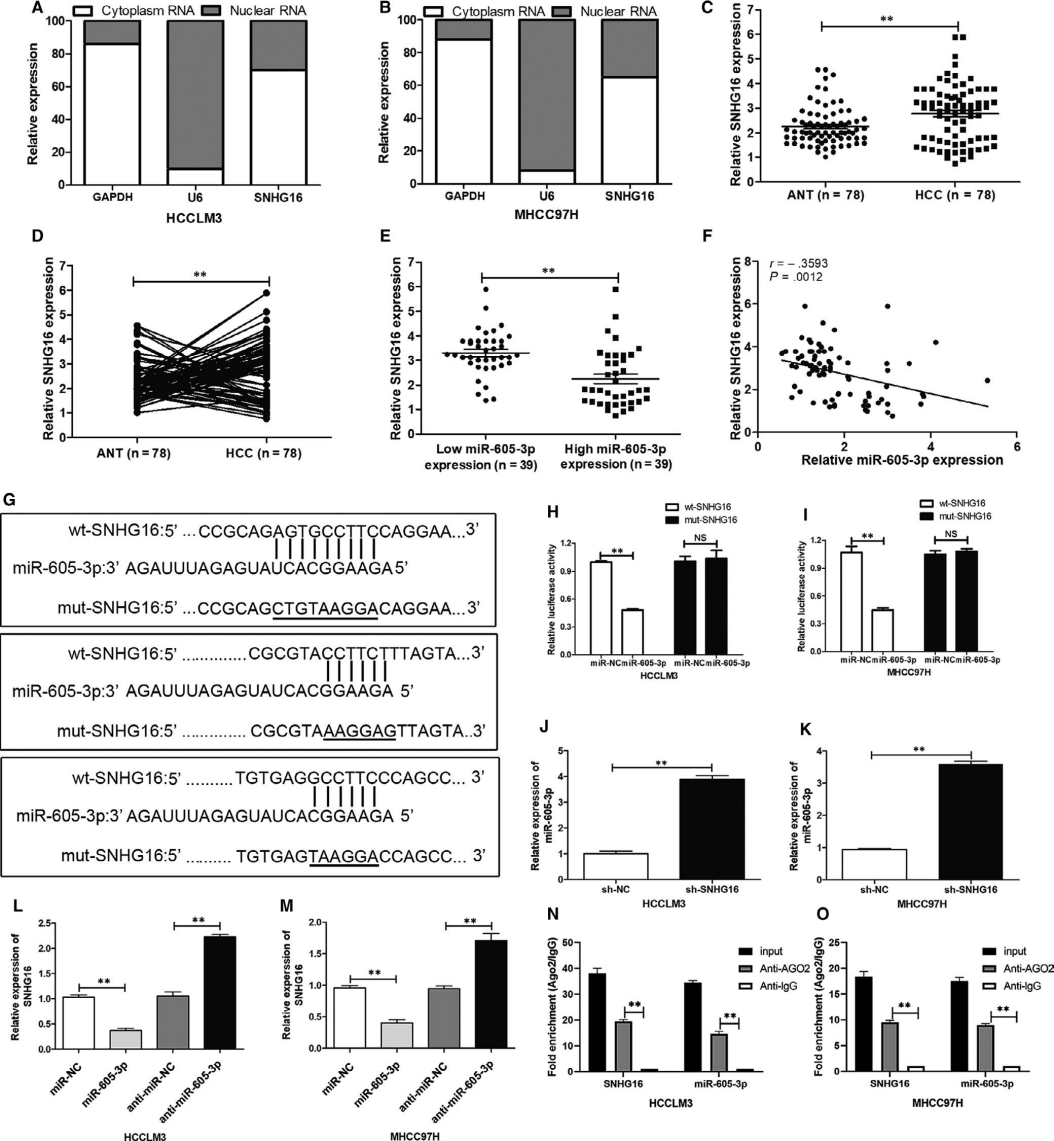
*SNHG16* directly binds to *miR‐605‐3p*. A and B, Cell fractionation and real‐time qPCR were performed to detect *SNHG16* cytoplasmic and nuclear expression levels in HCCLM3 and MHCC‐97H cells. *U6* was used as a nuclear control, and *GAPDH* was used as a cytoplasmic control. C and D, The relative expression of *SNHG16* was detected by real‐time qPCR in 78 HCC tissues and adjacent normal tissues. E and F, *SNHG16* expression was negatively related to the expression of *miR‐605‐3p*. G, Predicted potential *SNHG16* binding sites and mutated nucleotides in the potential binding sequence of *miR‐605‐3p* in *SNHG16*. H and I, Dual‐luciferase reporter assays were performed to detect the effects of *miR‐605‐3p* overexpression on the luciferase activity of reporters driven by WT and MUT *SNHG16* in HCCLM3 and MHCC‐97H cells. J and K, Effects of *SNHG16* silencing on *miR‐605‐3p* expression in HCCLM3 and MHCC‐97H cells. L and M, Effects of *miR‐605‐3p* silencing on *SNHG16* expression in HCCLM3 and MHCC‐97H cells. N, O, RNA‐binding protein immunoprecipitation assays with anti‐AGO2 antibodies were performed in HCCLM3 and MHCC‐97H cells transiently transfected with *miR‐605‐3p*. *P < .05, **P < .01. NS: no significance

Accumulating evidence has revealed that lncRNAs can function as ceRNAs for miRNAs. To determine whether any lncRNAs regulate *TRAF6* expression through *miR‐605‐3p*, we searched for potential lncRNAs that bind to *miR‐605‐3p* using an online bioinformatic tool (Starbase). The small nucleolar RNA host gene (SNHG) family caught our attention. Among the predicted SNHG family members, *SNHG16* expression was highest in HCC tissues and predominantly located in the cytoplasm of HCC cells (Figure S2A; Figure 5A, B). *SNHG16* expression was up‐regulated in the 78 HCC tissues compared with adjacent non‐tumour tissues (Figure 5C, D) as well as in HCC cell lines (Figure S2B) and in the liver HCC data set of TCGA database (Figure S2C). *SNHG16* expression was also negatively correlated with *miR‐605‐3p* expression in the 78 HCC tissues (Figure 5E, F). High *SNHG16* expression was not only positively associated with tumour diameter, tumour thrombus, presence of an envelope and tumour satellites in the 78 HCC patients (Table S1), but also served as an independent prognostic indicator for both OS and DFS (Figure S3A–D; Table S2).

To validate *miR‐605‐3p* as a target for *SNHG16*, dual‐luciferase reporter assays were performed (Figure 5G). The results showed that cotransfection of WT‐*SNHG16* and miR‐605‐3p in HCCLM3 cells (Figure 5H) and MHCC‐97H cells (Figure 5I) decreased luciferase activity compared with the control group. However, the luciferase activity in the MUT‐*SNHG16* group was not affected.

We further examined whether *SNHG16* expression was regulated by ectopic *miR‐605‐5p* expression or inhibition. The results showed that up‐regulation of *miR‐605‐3p* in HCCLM3 and MHCC‐97H cells down‐regulated *SNHG16* expression, while down‐regulation of *miR‐605‐3p* elevated *SNHG16* expression (Figure 5L, 5M). Furthermore, the expression of *miR‐605‐3p* was increased by *SNHG16* silencing (Figure 5J, 5K, Figure S2D, E).

To examine whether *SNHG16* could sponge *miR‐605‐3p* expression in an RISC‐dependent manner, RNA immunoprecipitation assays were conducted. *SNHG16* and *miR‐605‐3p* were more abundant in the AGO2 pellet compared with the IgG pellet (Figure 5N, 5O). These data indicated that *SNHG16* may act as a ceRNA by sponging *miR‐605‐3p*.

### 3.7 Down‐regulation of *SNHG16 *suppresses HCC metastasis, EMT and NF‐κB activation by interacting with *miR‐605‐3p *in vitro and in vivo

We found that *SNHG16* knockdown inhibited EMT and NF‐κB signalling activity in HCC cells (Figure S4A–R). When HCC cells were cotransfected with *miR‐605‐3p* and sh‐*SNHG16*, the effects of *SNHG16* silencing on HCC cell metastasis (Figure S5A–H), EMT and *TRAF6* expression (Figure S5I, J) were partly abolished. Additionally, the suppressive effect of *SNHG16* silencing on NF‐κB promoter luciferase reporter activity was partly reversed by *miR‐605‐3p* silencing (Figure 5K, L). These data showed that the effect of *SNHG16* on promoting HCC cell metastasis was mediated by *miR‐605‐3p*.

**FIGURE 6:**
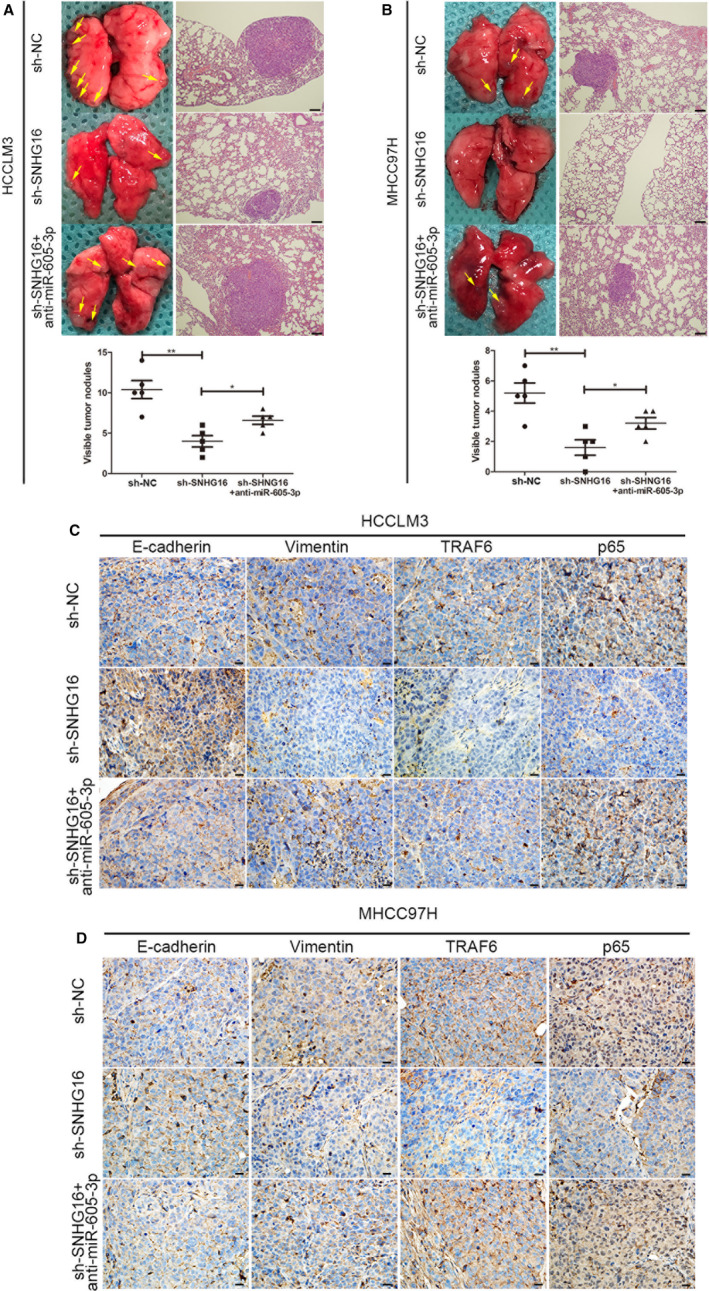
Down‐regulation of *SNHG16* inhibits HCC metastasis, EMT and NF‐κB signalling activation through *miR‐605‐3p *in vivo. A and B, (Left) Representative bright‐field images of lungs. (Right) Haematoxylin and eosin (H&E) staining of lung serial sections. Arrows indicate lung lesions. (Lower panel) Numbers of nodules on the lungs of mice (n = 5 per group) at 6 weeks after tail vein injection of HCCLM3/sh‐NC, HCCLM3/sh‐*SNHG16* or HCCLM3/sh‐*SNHG16*/anti‐*miR‐605‐3p* cells (scale bar, 25 μm) (A) or MHCC‐97H/sh‐NC, MHCC‐97H/sh‐*SNHG16* or MHCC‐97H/sh‐*SNHG16*/anti‐*miR‐605‐3p* (scale bar, 25 μm) (B). C and D, E‐cadherin, vimentin, TRAF6 and p65 staining in subcutaneous tumours from each group of nude mice injected with HCCLM3 cells (C) or MHCC‐97H cells (scale bar, 25 μm) (D). *P < .05, **P < .01

We then investigated whether *SNHG16* expression regulates the metastatic ability of HCC cells in vivo by interacting with *miR‐605‐3p*. HCCLM3/sh‐NC, HCCLM3/sh‐*SNHG16*, HCCLM3/sh‐*SNHG16*/anti‐miR‐605‐3p, MHCC‐97H/sh‐NC, MHCC‐97H/sh‐*SNHG16* or MHCC‐97H/sh‐*SNHG16*/anti‐miR‐605‐3p were injected into the tail vein of nude mice. We found that *SNHG16* silencing decreased the number and size of metastatic colonies from HCCLM3 cells (Figure 6A) and MHCC‐97H cells (Figure 6B) on the lung surface of mice. However, the effect of *SNHG16* silencing was partly abolished by *miR‐605‐3p* silencing.

A subcutaneously implanted tumour model in nude mice showed that *SNHG16* silencing decreased both the weight and growth rate of mice compared with mice injected with control HCCLM3 or MHCC‐97H cells (Figure S6A–E). The effects of *SNHG16* silencing in HCCLM3 and MHCC‐97H cells were partly reversed by *miR‐605‐3p* silencing. We examined E‐cadherin, vimentin, TRAF6 and p65 expression in the subcutaneous tumours by IHC and found that *SNHG16* silencing increased E‐cadherin expression, decreased vimentin expression, decreased TRAF6 expression and decreased p65 nuclear signals and that these effects were partly abolished by *miR‐605‐3p* silencing (Figure 6C, D).

### 3.8 *SNHG16* and NF‐κB form a positive feedback loop

**FIGURE 7:**
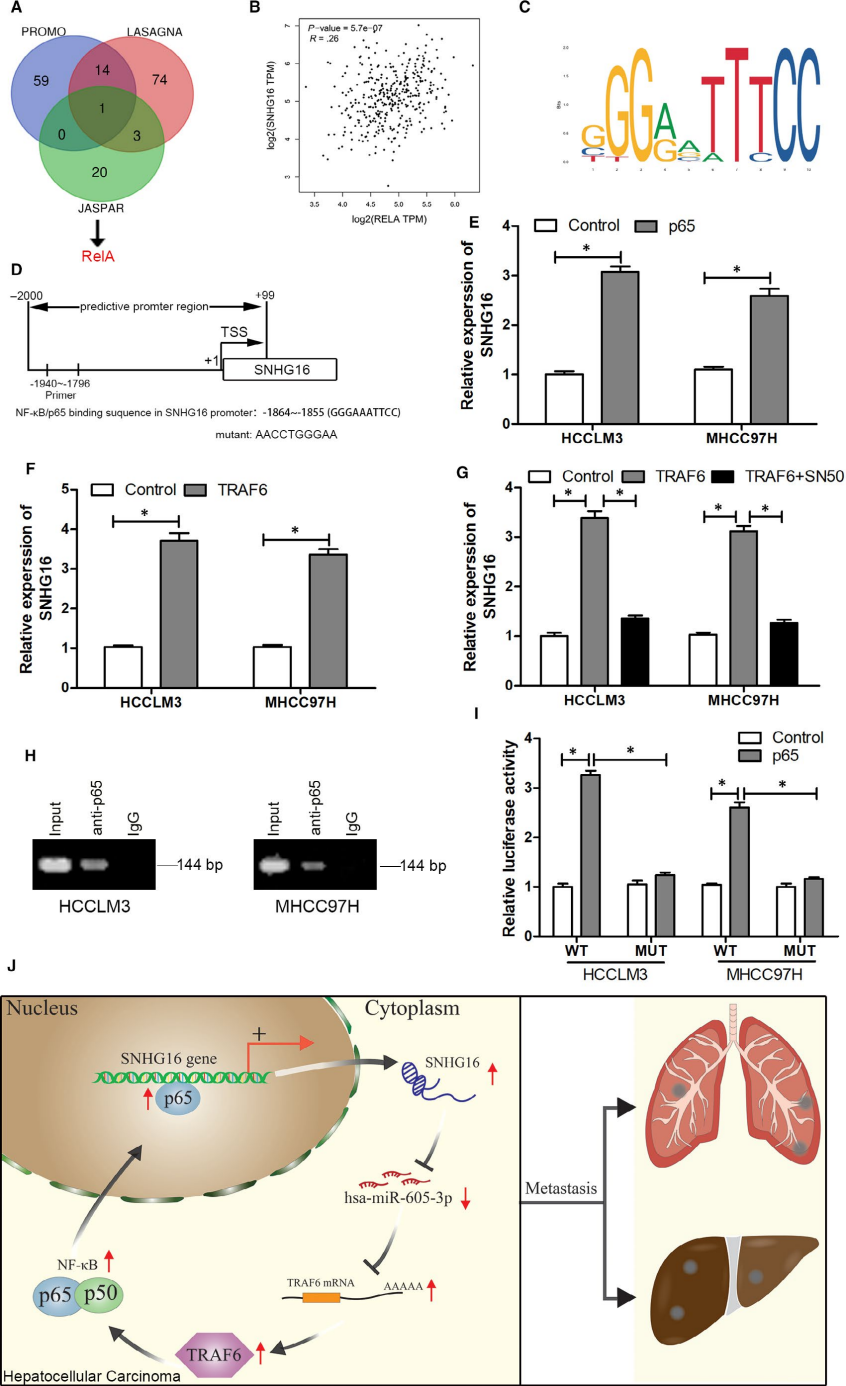
TRAF6 promotes *SNHG16* expression through NF‐κB signalling. A, Only RELA (NF‐κB/p65) was identified to possibly bind to the *SNHG16* gene promoter through the intersection of the JASPAR, PROMO and LASAGNA databases. B, Relative expression of *SNHG16* and NF‐κB/p65 in HCC was determined using the GEPIA database. C, JASPAR logo of NF‐κB/p65. D, Schematic diagram of the putative NF‐κB/p65‐binding sites in the *SNHG16* promoter. E, Real‐time qPCR was performed to determine the expression of *SNHG16* after NF‐κB/p65 overexpression in HCCLM3 and MHCC‐97H cells. F, Real‐time qPCR was performed to determine the expression of *SNHG16* after TRAF6 overexpression in HCCLM3 and MHCC‐97H cells. G, Real‐time qPCR was performed to determine the expression of *SNHG16* after TRAF6 overexpression in HCCLM3 and MHCC‐97H cells treated with or without SN‐50 (NF‐κB signalling inhibitor). H, ChIP assays were performed to detect NF‐κB/p65 on the *SNHG16* promoter in HCCLM3 and MHCC‐97H cells. I, Luciferase reporter assays were performed to confirm NF‐κB activation of the *SNHG16* promoter through the NF‐κB/p65‐binding sites in HCCLM3 and MHCC‐97H cells. J, Schematic illustration showing the positive feedback loop. *SNHG16* up‐regulates TRAF6 expression by directly binding to *miR‐605‐3p* as a ceRNA. TRAF6 promotes *SNHG16* expression by activating NF‐κB signalling. *P < .05, **P < .01

We further examined whether any of the identified factors act upstream of *SNHG16* in HCC. We analysed the promoter sequence of *SNHG16* using the PROMO, JASPAR and LASAGNA databases and found that RELA (NF‐κB/p65) was identified as a potential binding factor to the *SNHG16* gene promoter in all three databases (Figure 7A). We also found a positive correlation between the expression of *SNHG16* and NF‐κB/p65 in HCC using the GEPIA database (Figure 7B; *R* = .26, *P* < .001). These findings suggest that the promoter region of *SNHG16* may contain a binding motif for NF‐κB/p65 (Figure 7C, D). *SNHG16* expression was up‐regulated in HCCLM3 and MHCC‐97H cells in response to ectopic expression of NF‐κB/p65 compared with the empty vector group (Figure 7E). As NF‐κB is a downstream target of TRAF6, we examined whether TRAF6 overexpression significantly elevated *SNHG16* expression (Figure 7F). However, treatment with the NF‐κB inhibitor SN‐50 largely abolished the effect of TRAF6 on *SNHG16* expression (Figure 7G). We next examined whether NF‐κB/p65 interacted with promoter region of *SNHG16* via the predicted binding site. ChIP assays demonstrated that NF‐κB/p65 binds the *SNHG16* promoter (Figure 7H). Dual‐luciferase reporter assays showed that ectopic expression of NK‐κB/p65 enhanced luciferase activity driven by the WT‐*SNHG16* promoter (Figure 7I). When the NF‐κB‐binding sequence in the *SNHG16* promoter was mutated, luciferase expression was significantly decreased. These results indicate that NF‐κB/p65 interacted with the *SNHG16* promoter via the predicted binding site.

## 4 DISCUSSION

In the present study, we revealed that the putative tumour suppressor *miR‐605‐3p* was frequently silenced in HCC cell lines and tissues. Overexpression of *miR‐605‐3p* inhibited HCC cell metastasis in vitro and in vivo via the NF‐κB pathway. More importantly, low *miR‐605‐3p* expression was associated with malignant clinicopathological characteristics and poor survival outcome in HCC patients. Thus, examination of *miR‐605‐3p* expression by qRT‐PCR could be used as an additional tool for distinguishing HCC patients at high risk of metastasis and may provide useful information for clinicians to optimize individual therapy management for HCC.

We also determined the underlying mechanism involved in *miR‐605‐3p* regulation of the NF‐κB pathway. miRNAs play multiple cellular roles by targeting different genes. Bioinformatics analyses confirmed that TRAF6, a signal transducer for inflammatory NF‐κB signalling activation,^18^ is a direct target of *miR‐605‐3p* that affects HCC metastatic malignancy. TRAF6 acts as an E3 ubiquitin ligase that activates IκB kinase (IKK), which leads to IκBα degradation and p65 nuclear translocation, which suggests an important role for TRAF6 in NF‐κB activation.^19^, ^20^ Additionally, studies have shown that TRAF6 has an important role in the development of various cancers.^21^, ^22^, ^23^ Activation of signalling downstream of TRAF6, including NF‐κB, has a significant role in TRAF6‐mediated tumorigenesis.^24^ In the present study, we showed that TRAF6 participates in the abnormal expression of *miR‐605‐3p* and induces metastasis and EMT promotion in HCC. Co‐expression of *miR‐605‐3p* and TRAF6 largely reversed the tumour‐suppressive effects of *miR‐605‐3p* up‐regulation alone, which suggests that *miR‐605‐3p* inhibits the malignant behaviour of HCC cells by suppressing TRAF6 expression. Our findings also showed that *miR‐605‐3p* inhibited HCC metastasis by inhibiting NF‐κB activation and that this effect of *miR‐605‐3p* was dependent on TRAF6, which indicates that the *miR‐605‐3p*/*TRAF6*/NF‐κB axis functions in the regulation of HCC metastasis.

The ceRNA hypothesis proposes that numerous non‐coding RNAs may function as molecular sponges for miRNAs and thus functionally liberate RNA transcripts that are targeted by these miRNAs. Through bioinformatics analysis and PCR validation, *SNHG16* was determined to likely function as a ceRNA with *miR‐605‐3p* and affects the expression of TRAF6. *SNHG16* has been identified as an oncogene in many cancers.^25^, ^26^, ^27^ Up‐regulation of *SNHG16* predicts poor prognosis and induces sorafenib resistance in HCC.^28^ High *SNHG16* not only contributes to the promotion of HCC proliferation through the *miR‐302a‐3p*/FGF19 axis, but also sponges *miR‐4500* and targets STAT3, and sponges *miR‐195* to aggravate the tumorigenesis and development of HCC 29‐31. In line with these previous studies, our results suggest that *SNHG16* may function as an oncogene in HCC by promoting cell migration and invasion. To ascertain whether there was direct binding between *SNHG16* and *miR‐605‐3p*, we examined the subcellular localization of *SNHG16* by nucleoplasm separation and found that *miR‐605‐3p* and *SNHG16* were both mainly localized in the cytoplasm. Additionally, we conducted luciferase reporter assays and RNA‐IP analyses. We verified that *SNHG16* directly binds to *miR‐605‐3p* via a putative MRE and that the RISC was involved in this ceRNA regulatory network. Taken together, these results suggest that there is reciprocal repression between *SNHG16* and *miR‐605‐3p* mediated by the RISC and that *SNHG16* likely binds to other miRNAs as well as *miR‐605‐3p*.

One of the most interesting findings in this study was that NF‐κB can also regulate the expression of *SNHG16*. While *SNHG16* has been identified as an oncogene in many cancers including HCC,^28^, ^29^, ^30^, ^31^, ^32^, ^33^, ^34^ the biological functions of *SNHG16* and its underlying mechanisms in HCC are not fully understood. Positive feedback regulation is common in the regulation of many biological functions, especially sustained activation of cancer‐promoting signalling pathways.^35^ Multiple studies have shown that abnormal activation of the NF‐κB pathway occurs in various cancers.^36^, ^37^ However, the mechanism for its continued activation in HCC remains unclear. We found that *SNHG16* acts as a ceRNA to compete for *miR‐605‐3p*, thereby protecting *TRAF6* from *miR‐605‐3p* and ultimately activating the NF‐κB pathway. Zhou et al found that *SNHG16* expression was elevated in LPS‐induced WI‐38 cells, while Wang et al found that *SNHG16* reversed the effects of LPS.^38^, ^39^ Furthermore, LPS is known as an NF‐κB pathway activator.^40^ We speculated whether NF‐κB, as a transcription factor, could promote *SNHG16* expression by binding to its promoter region. Thus, we analysed the promoter sequence of *SNHG16* using the PROMO algorithm and JASPAR. The results revealed the presence of a putative binding site for NF‐κB within the *SNHG16* promoter region. Moreover, we found a positive correlation between the expression of *SNHG16* and p65 in HCC in the GEPIA database. Taken together with the ChIP and dual‐luciferase reporter assays, these results demonstrated that activation of the NF‐κB signalling pathway promotes *SNHG16* expression.

In summary, the present study demonstrates the tumour‐suppressive role of *miR‐605‐3p* in HCC metastasis for the first time and indicates the importance of the interactions between *SNHG16*, *miR‐605‐3p*, *TRAF6* and the NF‐κB pathway in the regulation of HCC cell malignancy. Decreased expression of *SNHG16* promotes *miR‐605‐3p* expression, which down‐regulates *TRAF6* and NF‐κB signalling and thereby inhibits a series of metastatic effects in HCC cells. In turn, activated NF‐κB can up‐regulate *SNHG16* expression to form a positive feedback loop. Thus, targeting the *SNHG16*/*miR‐605‐3p*/*TRAF6*/NF‐κB loop may be a potential new therapeutic strategy to improve the treatment and survival of HCC patients (Figure 7J).

## CONFLICT OF INTEREST

The authors confirm that there are no conflicts of interest.

## AUTHOR CONTRIBUTIONS

WJX and QSM conceived and designed the experiments. YLH, YF and YYC participated in the experiments and drafted the manuscript. YS and JZL contributed to the sample collection and interpretation of the data. PL and HH performed the statistical analyses. All authors read and approved the final manuscript.

## Supporting information

Fig S1

Fig S2

Fig S3

Fig S4

Fig S5

Fig S6

Table S1

Table S2

Table S3

Table S4

Supplementary Material

Supplementary Material

## Data Availability

The data sets used and/or analysed during the current study are available from the corresponding author on reasonable request.
