# Supplementary figures and images for: SNHG16/miR‐605‐3p/TRAF6/NF‐κB feedback loop regulates hepatocellular carcinoma metastasis
Source: J Cell Mol Med. 2020 May 20;24(13):7637–51. doi: 10.1111/jcmm.15399 (PMC7339162; doi:10.1111/jcmm.15399)

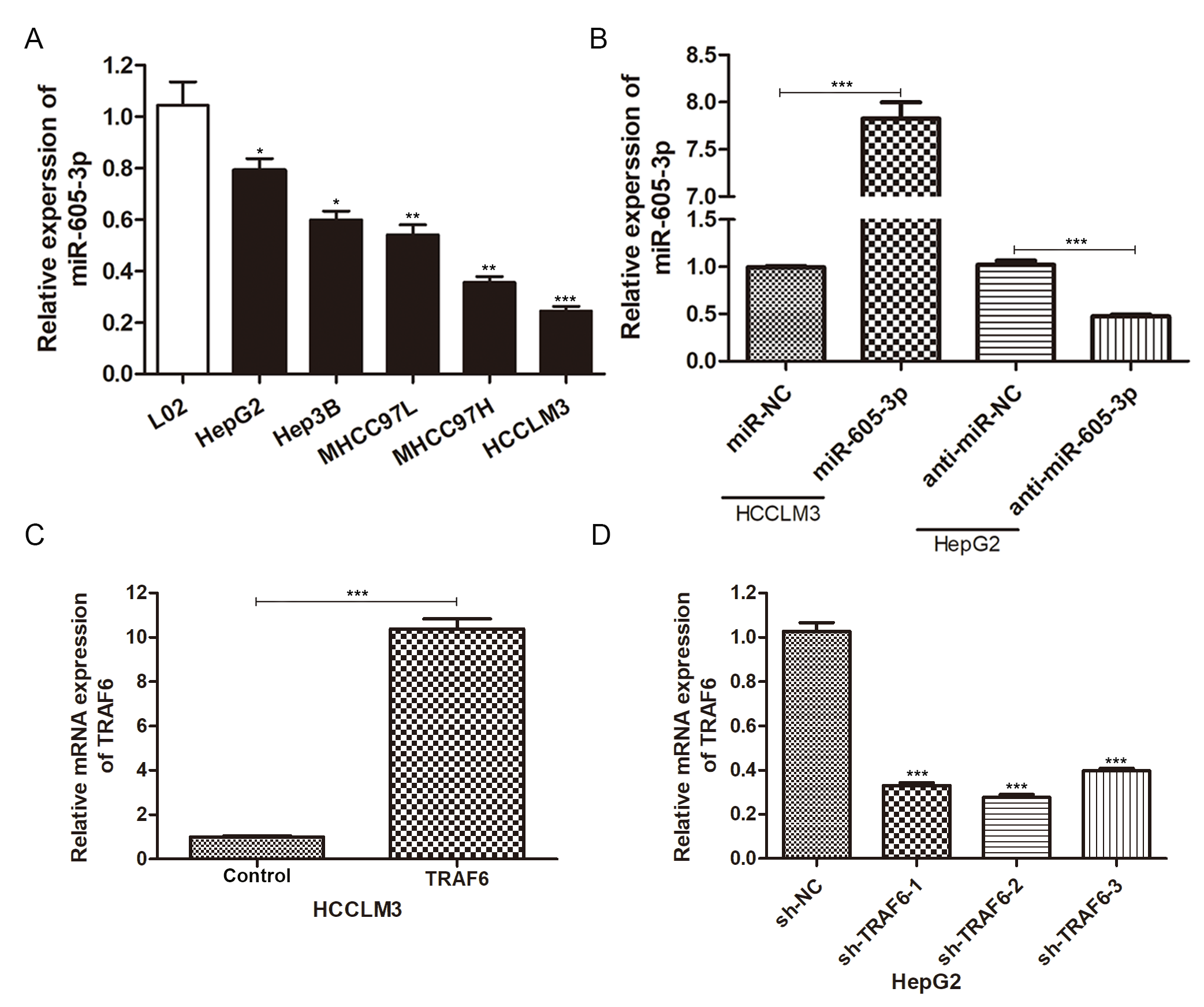

Supplement: Supplementary file 1 — Fig S1 [file JCMM-24-7637-s001.tif]

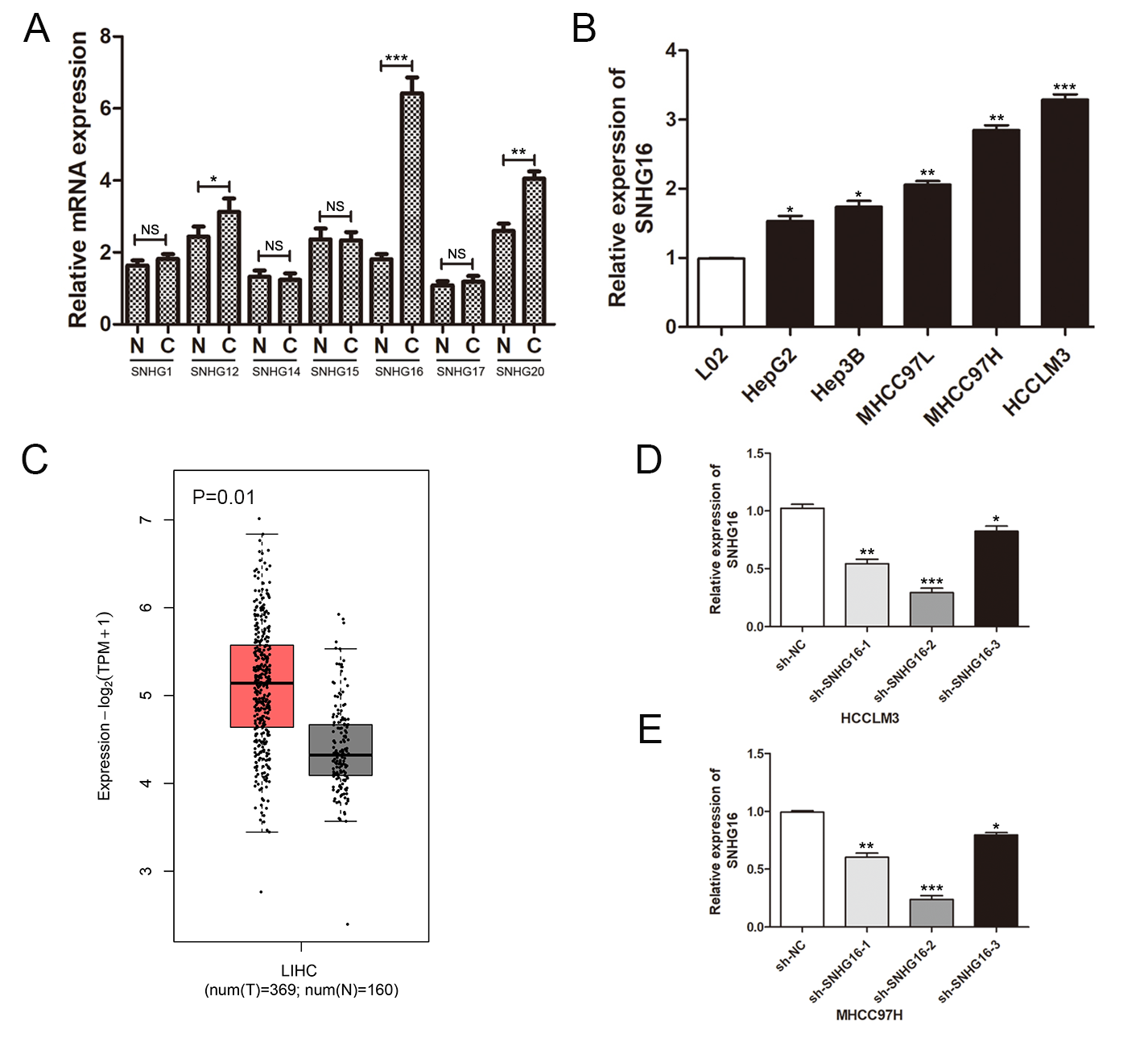

Supplement: Supplementary file 2 — Fig S2 [file JCMM-24-7637-s002.tif]

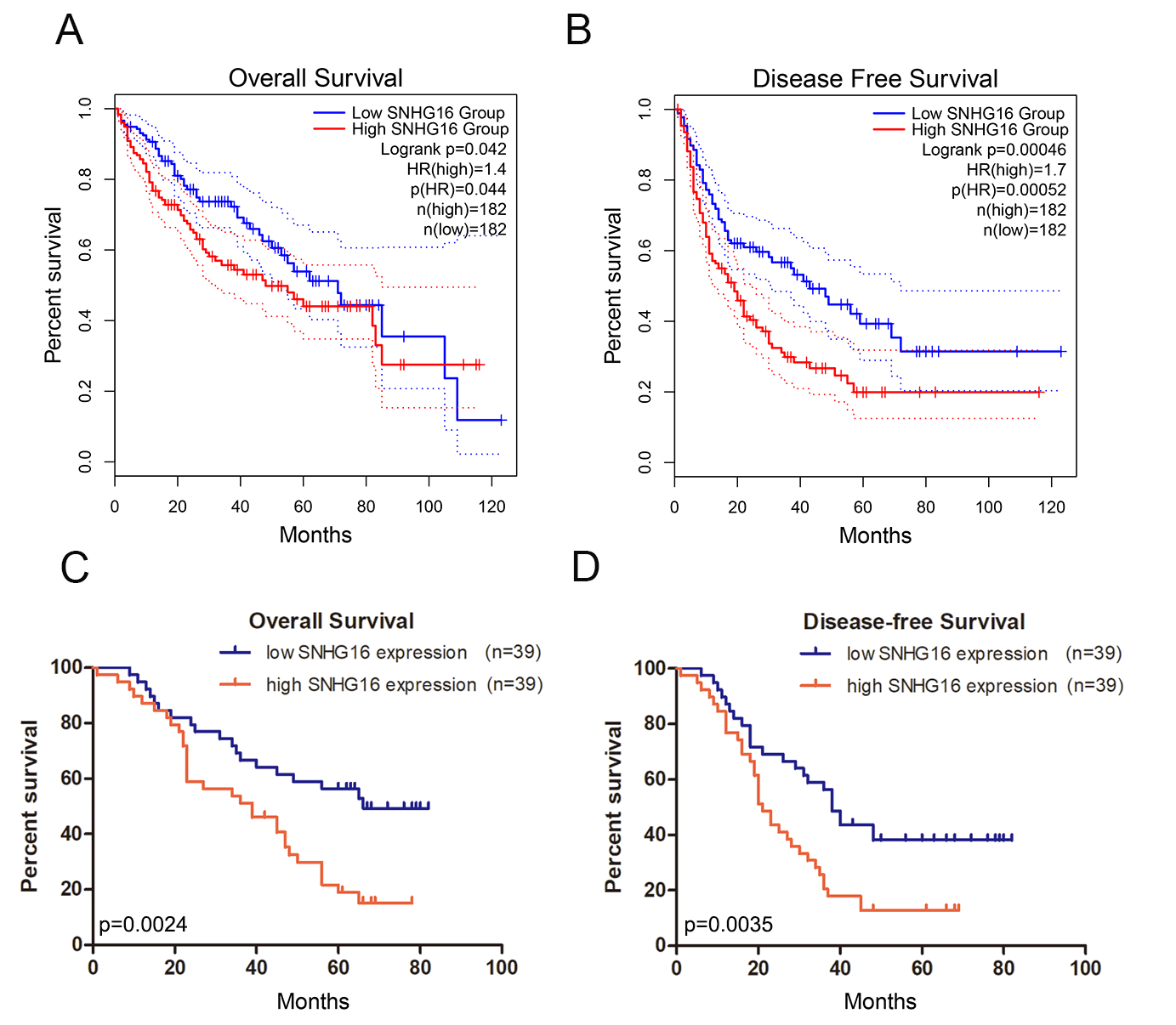

Supplement: Supplementary file 3 — Fig S3 [file JCMM-24-7637-s003.tif]

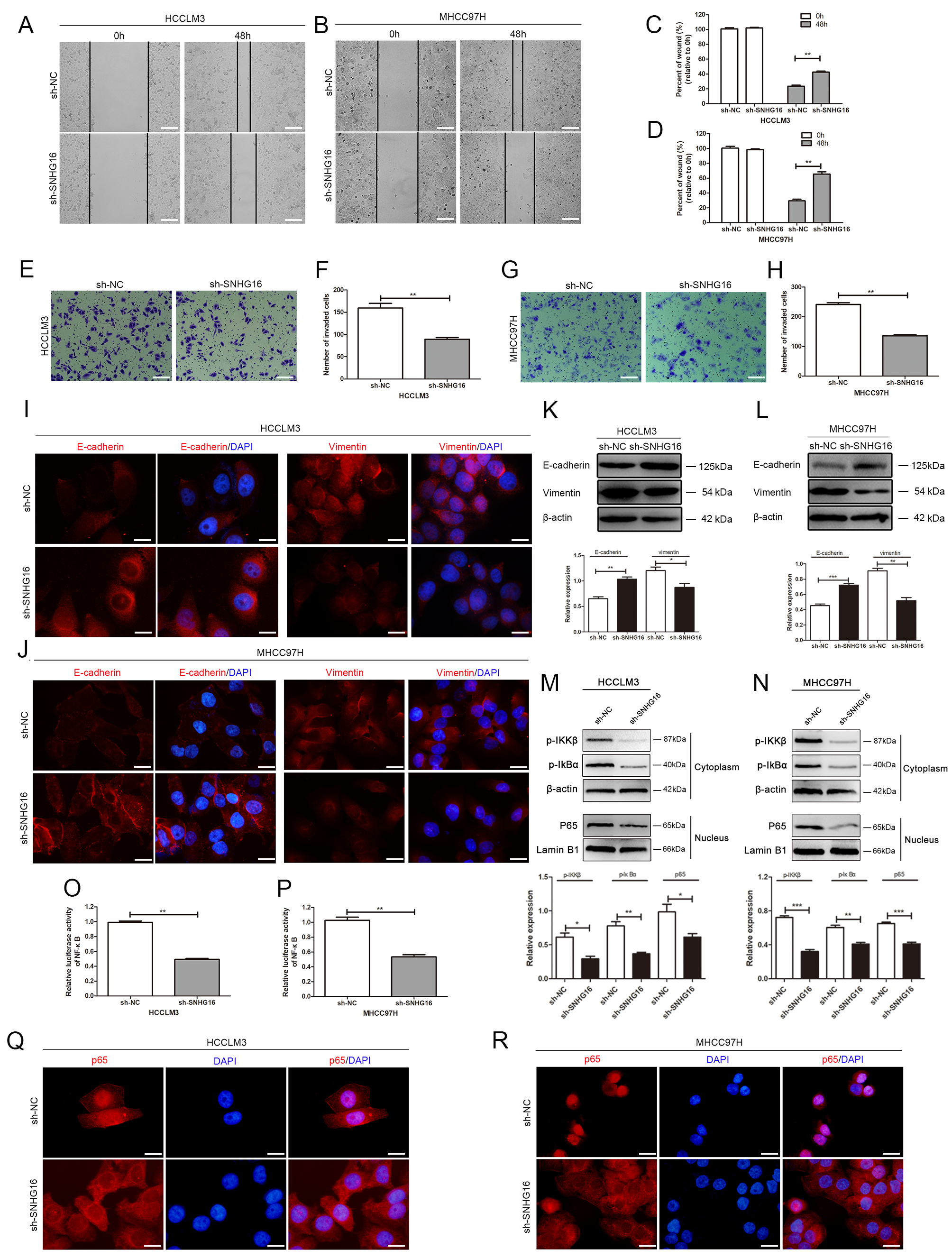

Supplement: Supplementary file 4 — Fig S4 [file JCMM-24-7637-s004.tif]

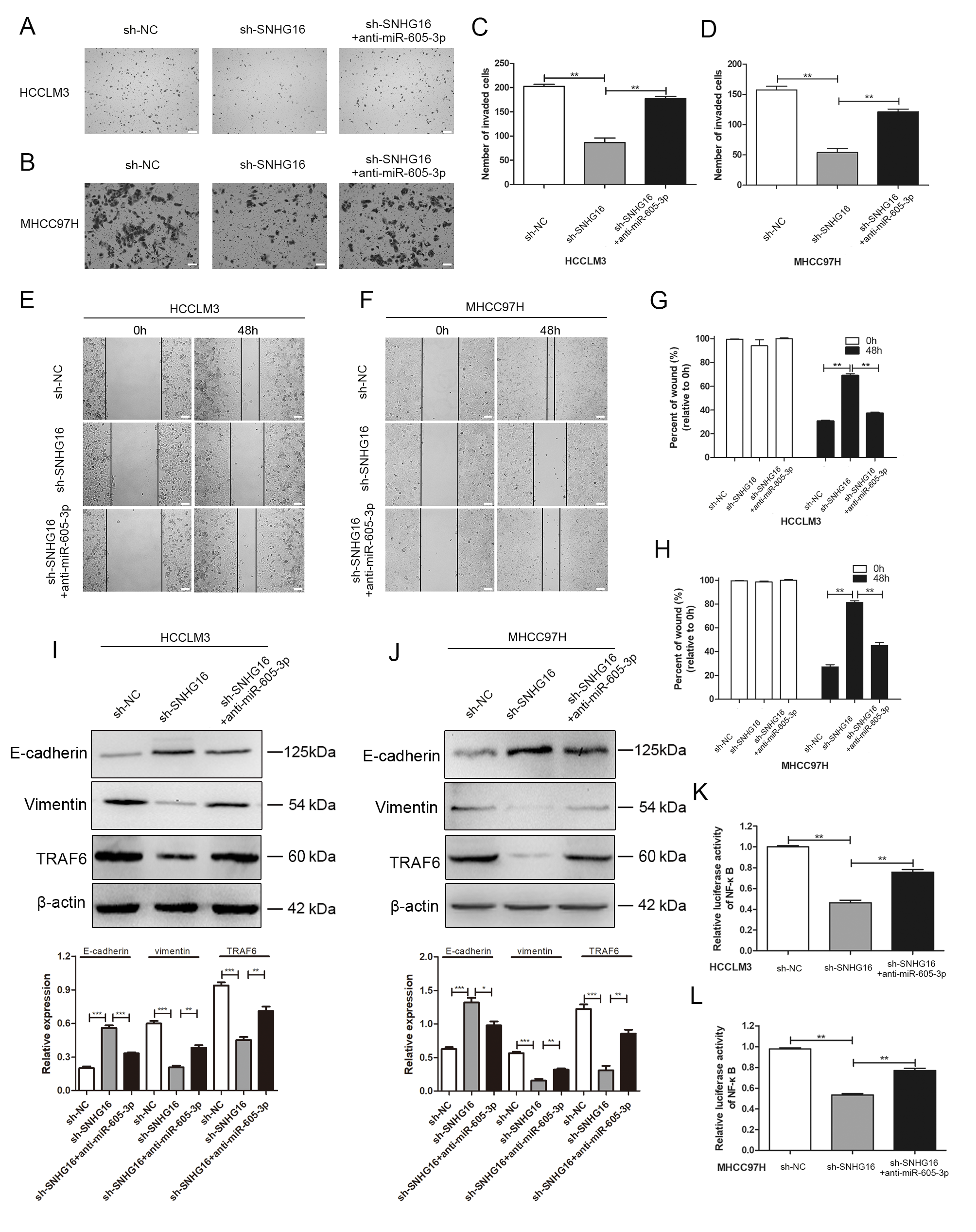

Supplement: Supplementary file 5 — Fig S5 [file JCMM-24-7637-s005.tif]

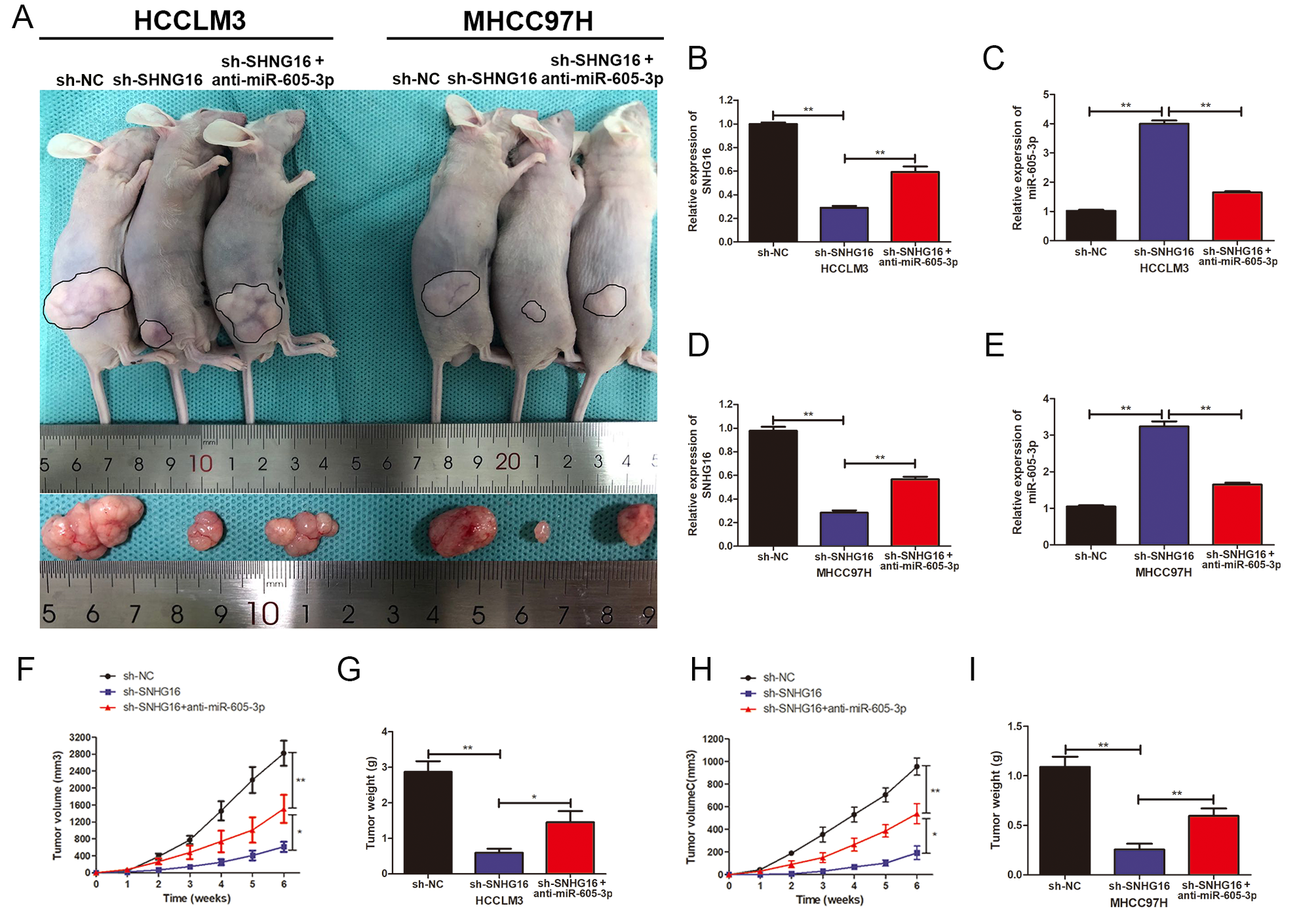

Supplement: Supplementary file 6 — Fig S6 [file JCMM-24-7637-s006.tif]
