# Supplementary material for: SNHG16/miR‐605‐3p/TRAF6/NF‐κB feedback loop regulates hepatocellular carcinoma metastasis
Source: J Cell Mol Med. 2020 May 20;24(13):7637–51. doi: 10.1111/jcmm.15399 (PMC7339162; doi:10.1111/jcmm.15399)
Supplement: Supplementary file 7 — Table S1 [file JCMM-24-7637-s007.doc]

**Table S1. Relationships between *SNHG16* expression and clinicopathological characteristics of HCC patients**

| **Clinicopathological characteristics** | **n** | **Low expression** | **High expression** | ***χ^2^*** | ***P* value** |
| --- | --- | --- | --- | --- | --- |
| **Total** | 78 | 39 | 39 |  |  |
| **Gender** |  |  |  | 2.6 | 0.107 |
| Male | 60 | 33 | 27 |  |  |
| Female | 18 | 6 | 12 |  |  |
| **Age (years)** |  |  |  | 0.051 | 0.821 |
| ≤54 | 41 | 21 | 20 |  |  |
| >54 | 37 | 18 | 19 |  |  |
| **Grade of differentiation** |  |  |  | 2.885 | 0.089 |
| Low | 25 | 9 | 16 |  |  |
| High-Middle | 53 | 30 | 23 |  |  |
| **Tumor diameter (cm)** |  |  |  | 11.661 | **0.001*** |
| ≤5 | 43 | 14 | 29 |  |  |
| >5 | 35 | 25 | 10 |  |  |
| **Liver function (Child-Pugh stage)** |  |  |  | 0.083 | 0.774 |
| A | 63 | 32 | 31 |  |  |
| B or C | 15 | 7 | 8 |  |  |
| **Hepatocirrhosis** |  |  |  | 3.692 | 0.055 |
| Absent | 26 | 9 | 17 |  |  |
| Present | 52 | 30 | 22 |  |  |
| **HBV infection** |  |  |  | 0.205 | 0.651 |
| Absent | 38 | 18 | 20 |  |  |
| Present | 40 | 21 | 19 |  |  |
| **Tumor thrombus** |  |  |  | 9.848 | **0.002*** |
| Absent | 66 | 38 | 28 |  |  |
| Present | 12 | 1 | 11 |  |  |
| **AFP (ng/ml)** |  |  |  | 0.206 | 0.65 |
| ≤20 | 42 | 20 | 22 |  |  |
| >20 | 36 | 19 | 17 |  |  |
| **BCLC stage** |  |  |  | 3.193 | 0.074 |
| A | 57 | 25 | 32 |  |  |
| B, C, or D | 21 | 14 | 7 |  |  |
| **Envelope** |  |  |  | 5.299 | **0.021*** |
| Absent | 46 | 18 | 28 |  |  |
| Present | 32 | 21 | 11 |  |  |
| **Tumor satellite** |  |  |  | 7.863 | **0.005*** |
| Absent | 62 | 36 | 26 |  |  |
| Present | 16 | 3 | 13 |  |  |

**P*<0.05. AFP: serum alpha fetoprotein; HBV: hepatitis B virus; BCLC: Barcelona Clinic Liver Cancer.
