# Supplementary material for: SNHG16/miR‐605‐3p/TRAF6/NF‐κB feedback loop regulates hepatocellular carcinoma metastasis
Source: J Cell Mol Med. 2020 May 20;24(13):7637–51. doi: 10.1111/jcmm.15399 (PMC7339162; doi:10.1111/jcmm.15399)
Supplement: Supplementary file 8 — Table S2 [file JCMM-24-7637-s008.doc]

**Table S2. Univariate and multivariable analyses of OS and DFS in HCC patients**

| **Variable** | **OS** | | | **DFS** | | |
| --- | --- | --- | --- | --- | --- | --- |
|  | **Univariate analysis** | **Multivariable analysis** | | **Univariate analysis** | **Multivariable analysis** | |
|  | ***P*>\|z\|** | ***P*>\|z\|** | **HR(95%CI)** | ***P*>\|z\|** | ***P*>\|z\|** | **HR(95%CI)** |
| **SNHG16 expression** |  |  |  |  |  |  |
| Low (n=39) vs. high (n=39) | **0.004*** | **0.024*** | 2.046(1.099-3.811) | **0.005*** | **0.032*** | 1.873(1.054-3.330) |
| **Gender** |  |  |  |  |  |  |
| Male (n=60) vs. female (n=18) | 0.131 |  |  | 0.205 |  |  |
| **Age (years)** |  |  |  |  |  |  |
| ≤54 (n=41) vs. >54 (n=37) | 0.833 |  |  | 0.537 |  |  |
| **Grade of differentiation** |  |  |  |  |  |  |
| Low (n=39) vs. middle-high (n=39) | 0.101 |  |  | 0.148 |  |  |
| **Tumor diameter (cm)** |  |  |  |  |  |  |
| ≤5 (n=43) vs. >5 (n=35) | 0.200 |  |  | 0.194 |  |  |
| **Liver function (Child-Pugh stage)** |  |  |  |  |  |  |
| A (n=63) vs. B or C (n=15) | 0.182 |  |  | 0.507 |  |  |
| **Hepatocirrhosis** |  |  |  |  |  |  |
| Absent (n=26) vs. present (n=52) | 0.425 |  |  | 0.176 |  |  |
| **Hepatitis B virus** |  |  |  |  |  |  |
| Absent (n=38) vs. present (n=40) | 0.177 |  |  | 0.553 |  |  |
| **Tumor thrombus** |  |  |  |  |  |  |
| Absent (n=66) vs. present (n=12) | **0.011*** |  |  | **0.008*** |  |  |
| **AFP (ng/ml)** |  |  |  |  |  |  |
| ≤20 (n=42) vs. >20 (n=36) | 0.060 |  |  | 0.107 |  |  |
| **BCLC stage** |  |  |  |  |  |  |
| I (n=57) vs. II, III, or IV (n=21) | 0.151 |  |  | 0.763 |  |  |
| **Envelope** |  |  |  |  |  |  |
| Absent (n=46) vs. present (n=32) | 0.189 |  |  | 0.168 |  |  |
| **Tumor satellite** |  |  |  |  |  |  |
| Absent (n=62) vs. present (n=16) | 0.068 |  |  | 0.069 |  |  |

**P*<0.05.
