# Supplementary material for: SNHG16/miR‐605‐3p/TRAF6/NF‐κB feedback loop regulates hepatocellular carcinoma metastasis
Source: J Cell Mol Med. 2020 May 20;24(13):7637–51. doi: 10.1111/jcmm.15399 (PMC7339162; doi:10.1111/jcmm.15399)
Supplement: Supplementary file 9 — Table S3 [file JCMM-24-7637-s009.doc]

**Table S3. Primers used for reverse transcription and real-time PCR**

| **Primer names** | **Sequences** |
| --- | --- |
| SNHG1 | F: ACGTT GGAACCGAAGAGAGC |
|  | R: GCAGCTGAA TTCCCCAGGAT |
| SNHG12 | F: TCTGGTGATCGAGGACTTCC |
|  | R: ACCTCCTCAGTATCACACACT |
| SNHG14 | F: GGGTGTTTACGTAGACCAGAACC |
|  | R: CTTCCAAAAGCCTTCTGCCTTAG |
| SNHG15 | F: CCGGCCAGGAATAGTTGCTGAA |
|  | R: ACTGGTGAGAGGTGGCTGTCA |
| SNHG16 | F: AGCCATGTAGAACTGTAAGTCCAA |
|  | R: CAGAGTTACTGGCACGAGGACA |
| SNHG17 | F: GTTCCTGGGGCTTGGATGAT |
|  | R: GATCTAAGGCTGAGACCCACG |
| SNHG20 | F: ATGGCTATAAATAGATACACG |
|  | R: GGTACAAACAGGGAGGGA |
| TRAF6 | F: TTTGCTCTTATGGATTGTCCCC |
|  | R: CATTGATGCAGCACAGTTGTC |
| GAPDH | F: TGCACCACAACTGCTTAGC |
|  | R: GGCATGGACTGTGGTCATGAG |
| U6 | F: ATTGGAACGATACAGAGAAGATT |
|  | R: GGAACGCTTCACGAATTT G |
