# Supplementary material for: SNHG16/miR‐605‐3p/TRAF6/NF‐κB feedback loop regulates hepatocellular carcinoma metastasis
Source: J Cell Mol Med. 2020 May 20;24(13):7637–51. doi: 10.1111/jcmm.15399 (PMC7339162; doi:10.1111/jcmm.15399)
Supplement: Supplementary file 10 — Table S4 [file JCMM-24-7637-s010.doc]

**Table S4. Target sequences**

| Name | Target Seq |
| --- | --- |
| sh-TRAF6#1 | CACGGGAAATATGTAATAT |
| sh-TRAF6#2 | GAATTTCCAGGAAACTATT |
| sh-TRAF6#3 | CATCTGCTTGATGGCATTA |
| sh-SNHG16#1 | GGTAGCTGCTTTAGCAGTT |
| sh-SNHG16#2 | GGAATGAAGCAACTGAGAT |
| sh-SNHG16#3 | CCCAGTGTTGACTCACCAA |
| miR-605-3p agomir | AGAAGGCACUAUGAGAUUUAGA  UAAAUCUCAUAGUGCCUUCUUU |
| miR-605-3p antagomir | UCUAAAUCUCAUAGUGCCUUCU |
