# Supplementary material for: SNHG16/miR‐605‐3p/TRAF6/NF‐κB feedback loop regulates hepatocellular carcinoma metastasis
Source: J Cell Mol Med. 2020 May 20;24(13):7637–51. doi: 10.1111/jcmm.15399 (PMC7339162; doi:10.1111/jcmm.15399)
Supplement: Supplementary file 11 — Supplementary Material [file JCMM-24-7637-s011.docx]

**Supplementary Figure 1. *MiR-605-3p* expression is downregulated in HCC cells. a**, Relative expression of *miR-605-3p* in normal hepatic cells and five HCC cell lines by real-time qPCR. **b**, Relative expression of *miR-605-3p* in HCC cells after overexpression or silencing of *miR-605-3p*. **c**, **d**, Relative expression of *TRAF6* in HCC cells after overexpression or silencing of *TRAF6*. **P*<0.05, ** *P*<0.01, ****P*<0.001.

**Supplementary Figure 2. *SNHG16* expression is upregulated in HCC cells and HCC tissues. a**, Expression of SNHG family members in 16 HCC tissues and adjacent normal tissues were analyzed by real-time qPCR. **b**, *SNHG16* expression was analyzed in a normal hepatic cell line (L02) and five HCC cell lines by real-time qPCR. **c,** *SNHG16* expression was analyzed in the LIHC dataset of the TCGA database. **d**, **e**, The efficiency of *SNHG16* knockdown was detected in HCCLM3 and MHCC-97H cells. **P*<0.05, ** *P*<0.01, ****P*<0.001, NS: no significance.

**Supplementary Figure 3. High** **expression of *SNHG16* is related to poor prognosis of HCC patients.** **a**, **b**, Correlation between *miR-605-3p* expression and OS (**a**) and DFS (**b**) in the LIHC dataset of the TCGA database. **c**, **d**, Correlation between miR-605-3p expression and OS (**c**) and DFS (**d**) in the Nantong University Afﬁliated Hospital cohort.

**Supplementary Figure 4. Downregulation of *SNHG16* inhibits HCC metastasis, EMT, and NF-κB signaling activation. a–h**, The effects of *SNHG16* silencing on metastasis were detected by wound healing and matrigel transwell assays in HCCLM3 cells (**a**, **c**, **e**, **f**) and MHCC-97H cells (**b**, **d**, **g**, **h**) (scale bar, 100 μm). **i–l**, The effects of *SNHG16* silencing on EMT were detected by immunofluorescence and western blotting analyses in HCCLM3 cells (**i**, **k**) and MHCC-97H cells (scale bar, 25μm) (**j**, **l**). **o–r**, The effects of *SNHG16* silencing on NF-κB signaling activation were detected by NF-κB luciferase reporter activity and immunofluorescence assays in HCCLM3 cells (**o**, **q**) and MHCC-97H cells (scale bar, 25μm) (**p**, **r)**. **P*<0.05, ***P*<0.01.

**Supplementary Figure 5. Downregulation of *SNHG16* inhibits HCC metastasis, EMT, and NF-κB signaling activation through *miR-605-3p* in vitro. a–h**, Matrigel invasion and wound healing assays were performed to detect the invasion and migration ability in HCCLM3 cells (**a**, **c, e, g**) and MHCC-97H cells (**b**, **d, f, h**) transfected with sh-NC or sh-SNHG16 or co-transfected with sh-SNHG16 and anti-miR-605-3p (scale bar, 100 μm). **i**, **j**, Western blotting analyses were performed to detect the protein expression of EMT biomarkers and TRAF6 in HCCLM3 cells (**i**) and MHCC-97H cells (**j**). **k**, **l**, NF-κB luciferase reporter assays were performed to detect the activation of NF-κB signaling in HCCLM3 cells (**k**) and MHCC-97H cells (**l**). **P*<0.05, ***P*<0.01.

**Supplementary Figure 6.** **Downregulation of *SNHG16* inhibits HCC growth through miR-605-3p in vivo. a**, Representative images of HCC tumors from nude mice inoculated with HCCLM3/sh-NC, HCCLM3/sh-SNHG16, or HCCLM3/sh-SNHG16/anti-miR-605-3p cells and MHCC-97H/sh-NC, MHCC-97H/sh-SNHG16, or MHCC-97H/sh-SNHG16/anti-miR-605-3p cells. **b–e**, qRT-PCR analyses were performed to detect *SNHG16* and *miR-605-3p* expression in subcutaneous tumors. **f–i**, Growth curves for tumor volume and tumor weight were calculated. **P*<0.05, ***P*<0.01.
