# Supplementary material for: SNHG16/miR‐605‐3p/TRAF6/NF‐κB feedback loop regulates hepatocellular carcinoma metastasis
Source: J Cell Mol Med. 2020 May 20;24(13):7637–51. doi: 10.1111/jcmm.15399 (PMC7339162; doi:10.1111/jcmm.15399)
Supplement: Supplementary file 12 — Supplementary Material [file JCMM-24-7637-s012.docx]

**Supplementary Materials and Methods**

**RNA extraction and qRT-PCR**

Total RNA was extracted from HCC tissues and cell lines using Trizol (Invitrogen), and evaluated for quality and quantity by spectrophotometry. The total RNA was reverse-transcribed into cDNA using a cDNA Synthesis Kit (Roche). PCR amplification was performed with a SYBR Green I Master Kit (Roche), by incubation at 95℃ for 10 min followed by 40 cycles of 95℃ for 15 s, 60℃ for 32 s, and 72℃ for 32 s in an ABI 7500 PCR System (Applied Biosystems, Foster City, CA). The primer sequences are listed in Supplementary Table S3. *GAPDH* and *U6* were used as internal controls. All primers were purchased from Sangon Biotech (Shanghai, China). miRNA quantification was performed using Bulge-loop™ miRNA qRT-PCR Primer Sets (one RT primer and a pair of qPCR primers per set) specific for *miR-605-3p* as designed by RiboBio (Guangzhou, China).

**RNA FISH**

A *miR-605-3p* FISH Kit was purchased from GenePharma (Shanghai, China) and the experiment was performed according to the manufacturer’s instructions. The results were visualized with an IX71 inverted microscope (Olympus).

**Subcellular fractionation**

Nuclear and Cytoplasmic Protein Extraction Kit (Sangon Biotech) and Cytoplasmic & Nuclear RNA Purification Kit (Norgen Biotek, Canada) were used for subcellular fractionation in accordance with the manufacturer’s instructions. Lamin B1 and *U6* was used as a nuclear positive control, and *β-actin* was used a cytoplasmic positive control.

**Western blotting**

RIPA buffer was used to extract cell and tissue proteins with ProtLytic Phosphatase Inhibitor Cocktail (NCM Biotech, Soochow, China), followed by centrifugation at 10,000 × *g* for 30 min. After quantification with a BCA Protein Assay Kit (Beyotime, Shanghai, China), the total cellular protein extracts (30 μg/lane) were separated by sodium dodecyl sulfate-polyacrylamide gel electrophoresis and transferred to nitrocellulose membranes. The membranes were blocked with 5% BSA for 2 h at room temperature, and incubated overnight at 4°C with the following antibodies: anti-NF-κB p65 (Cell Signaling Technology, Danvers, MA; 8242), anti-p-IκBα (Cell Signaling Technology; 14D4), anti-p-IKKβ (Abcam; ab59195), anti-E-cadherin (Cell Signaling Technology; 24E10), anti-vimentin (Proteintech, Wuhan, China; 10366-1-AP), anti-TRAF6 (Abcam; ab137452), anti-lamin B1 (Proteintech; 66095-1-Ig), and anti-β-actin (Proteintech; 66009-1-Ig). The membranes were washed and incubated with appropriate horseradish peroxidase-conjugated secondary antibodies (MultiSciences, Wuhan, China) at room temperature for 2 h. Finally, signals were detected using Immobilon^TM^ Western Chemiluminescent HRP Substrate (Millipore). All experiments were performed in triplicate.

**Immunohistochemistry (IHC)**

IHC was performed in accordance with a previously described protocol 1. Staining intensity was scored as follows: 0, negative; 1, weakly positive; 2, moderately positive; and 3, strongly positive. The percentage of positive cells was also assessed according to four scores: 1 (0%–10%); 2 (11%–50%); 3 (51%–80%); and 4 (81%–100%). The degree of E-cadherin, vimentin, TRAF6 or p65 final IHC score was then generated by multiplying the intensity score with the percentage of positive cells (range from 0 to 3).

**Cell transfection, lentivirus production and transduction**

*TRAF6* ectopic-expressing and empty vector lentiviruses were purchased from GeneChem and was transfected into HCCLM3 cells. Short-hairpin RNA (shRNA) sequences targeting the *TRAF6* lentiviruses was transfected into HepG2 cells. Lentiviral particles containing shRNA sequences targeting *SNHG16* and negative control lentiviruses containing a random shRNA sequence were transfected into HCCLM3 and MHCC-97H cells. The shRNA sequences are listed in Supplementary Table S4. The *miR-605-3p* agomir (miR-650-3p) and negative control (miR-NC), *miR-605-3p* antagomir (anti-miR-605-3p) and negative control (anti-miR-NC) were purchased from Genepharma. The target sequences are listed in Supplementary Table S4. The efficiencies of RNA interference and ectopic expression were determined by qRT-PCR. The highest efficiency of were chose for following experiments (sh-TRAF6-2, sh-SNHG16-2).

**Wound healing assay**

HCC cells were seeded on 6-well plates, grown to confluency, and scratched with a 200-µl tip. Wound recovery was observed under an IX71 inverted microscope (Olympus) after 0 h and 48 h.

**Matrigel invasion assay**

Cells were seeded on 24-well transwell plates (Corning, New York, NY) to measure their invasive capacity. Inserts were coated with 60 µl of Matrigel (BD Biosciences, Franklin Lakes, NJ). After 24 h, invaded cells were fixed and stained with 0.1% crystal violet solution containing formaldehyde. The numbers of invaded cells were counted in five randomly-selected fields under an IX71 inverted microscope (Olympus).

**Immunofluorescence**

Cells grown on glass coverslips were fixed, permeabilized, blocked, and incubated overnight at 4° with the following primary antibodies: anti-E-cadherin (Cell Signaling Technology; 24E10), anti-vimentin (Proteintech; 10366-1-AP), and anti-NF-κB p65 (Cell Signaling Technology; 8242). The cells were then washed and incubated with Alexa Fluor 594-conjugated goat anti-rabbit IgG (ABclonal, Wuhan, China; AS039) for HCCLM3 and MHCC-97H cells or DyLight-488 goat anti-mouse IgG (MultiSciences; GAM4882) for HepG2 cells. Finally, the cells were counterstained with 4'-6-diamidino-2-phenylindole (DAPI) and imaged under a BX41 microscope (Olympus).

**RNA immunoprecipitation (RIP) assays**

RIP assays were conducted using the Magna RIP Kit (Millipore, Billerica, MA), according to the manufacturer’s protocol. Anti-AGO2 antibody and normal IgG (MultiSciences) were used for immunoprecipitation. The coprecipitated RNA was purified and analyzed by qPCR to assess the enrichment of *SNHG16* and *miR-605-3p*.

**In vivo animal experiments**

For tumor growth assays, five mice were included into each group. HCCLM3/sh-NC, HCCLM3/sh-SNHG16 and HCCLM3/sh-SNHG16/anti-miR-605-3p or MHCC-97H/sh-NC, MHCC-97H/sh-SNHG16 and MHCC-97H/sh-SNHG16/anti-miR-605-3p cells at 5 × 10^6^ cells/200 μl were subcutaneously injected into the right groin area of nude mice. After 6 weeks, the mice were euthanized, and the individual tumors were removed and weighed. Tumor growth was examined every 7 days and tumor volumes were calculated (0.5 × length × width^2^).

For tumor metastasis assays, five mice were included into each group. HCCLM3/sh-NC, HCCLM3/sh-SNHG16 and HCCLM3/sh-SNHG16/anti-miR-605-3p or MHCC-97H/sh-NC, MHCC-97H/sh-SNHG16 and MHCC-97H/sh-SNHG16/anti-miR-605-3p cells at 100 μl of PBS containing 5 × 10^6^ HCC cells was injected into nude mice via the tail vein. After 6 weeks, the mice were euthanized, and the metastatic nodules in each lung were analyzed. All animal experiments were performed using protocols approved by the Department of Animal Center, Medical College of Nantong University.

**Statistical analysis**

Each experiment was performed as least three times, and data were presented as mean ± SEM. Statistical analyses were performed using SPSS Version 24.0 software (SPSS, Chicago, IL). A value of *P* <0.05 was considered to indicate statistical significance. The χ^2^ test was used to determine the significance of differences in multiple comparisons. Survival curves were estimated by Kaplan–Meier analysis and compared by the log-rank test. Factors shown to be of prognostic significance in the univariate Cox regression model were subsequently investigated by the multivariate Cox regression model.

**REFERENCES**

1. Zhang JX, Chen ZH, Xu Y, et al. Downregulation of MicroRNA-644a Promotes Esophageal Squamous Cell Carcinoma Aggressiveness and Stem Cell-like Phenotype via Dysregulation of PITX2. *Clin Cancer Res*. 2017;23(1):298-310.
